# Dietary Modulation of microRNAs as a Potential Nutritional Strategy in Inflammatory and Autoimmune Skin Diseases: A Narrative Review

**DOI:** 10.3390/cells15181640

**Published:** 2026-09-10

**Authors:** Anna Czaplicka, Klaudia Dopytalska, Maciej Dawid, Elżbieta Szymańska, Irena Walecka

**Affiliations:** 1 Department of Dermatology, The National Institute of Medicine of the Ministry of the Interior and Administration, 02-507 Warsaw, Poland; 2 Department of Dermatology, Centre of Postgraduate Medical Education, 01-813 Warsaw, Poland

**Keywords:** atopic dermatitis, diet, epigenetic, microRNA, psoriasis

## Abstract

Inflammatory and autoimmune skin diseases like psoriasis, atopic dermatitis, and hidradenitis suppurativa present significant therapeutic challenges due to their complex immunological and environmental pathogenesis. This review highlights the critical role of microRNAs (miRNAs) as epigenetic regulators of inflammatory processes and investigates the potential of nutrigenomics to modulate their expression. Bioactive dietary components—including polyphenols such as quercetin, resveratrol, and curcumin, alongside vitamins C and D, selenium, and omega-3 fatty acids—demonstrate the ability to alter miRNA profiles like miR-21, miR-146a, and miR-155. These miRNA alterations may contribute to the regulation of key inflammatory pathways, including nuclear factor kappa-light-chain-enhancer of activated B cells (NF-κB) and mitogen-activated protein kinase (MAPK), thereby potentially influencing pro-inflammatory cytokine production and keratinocyte proliferation. However, the extent to which individual dietary compounds directly mediate effects on these pathways through miRNA regulation varies and is often supported mainly by preclinical evidence. Clinical data suggests that dietary interventions, such as weight loss and the Mediterranean diet alleviate skin symptoms and lower systemic inflammatory markers. Identifying disease-specific miRNA expression profiles represents an emerging research avenue that may, following analytical and clinical validation, inform future approaches in dermatology.

## 1. Introduction

**Figure 1:**
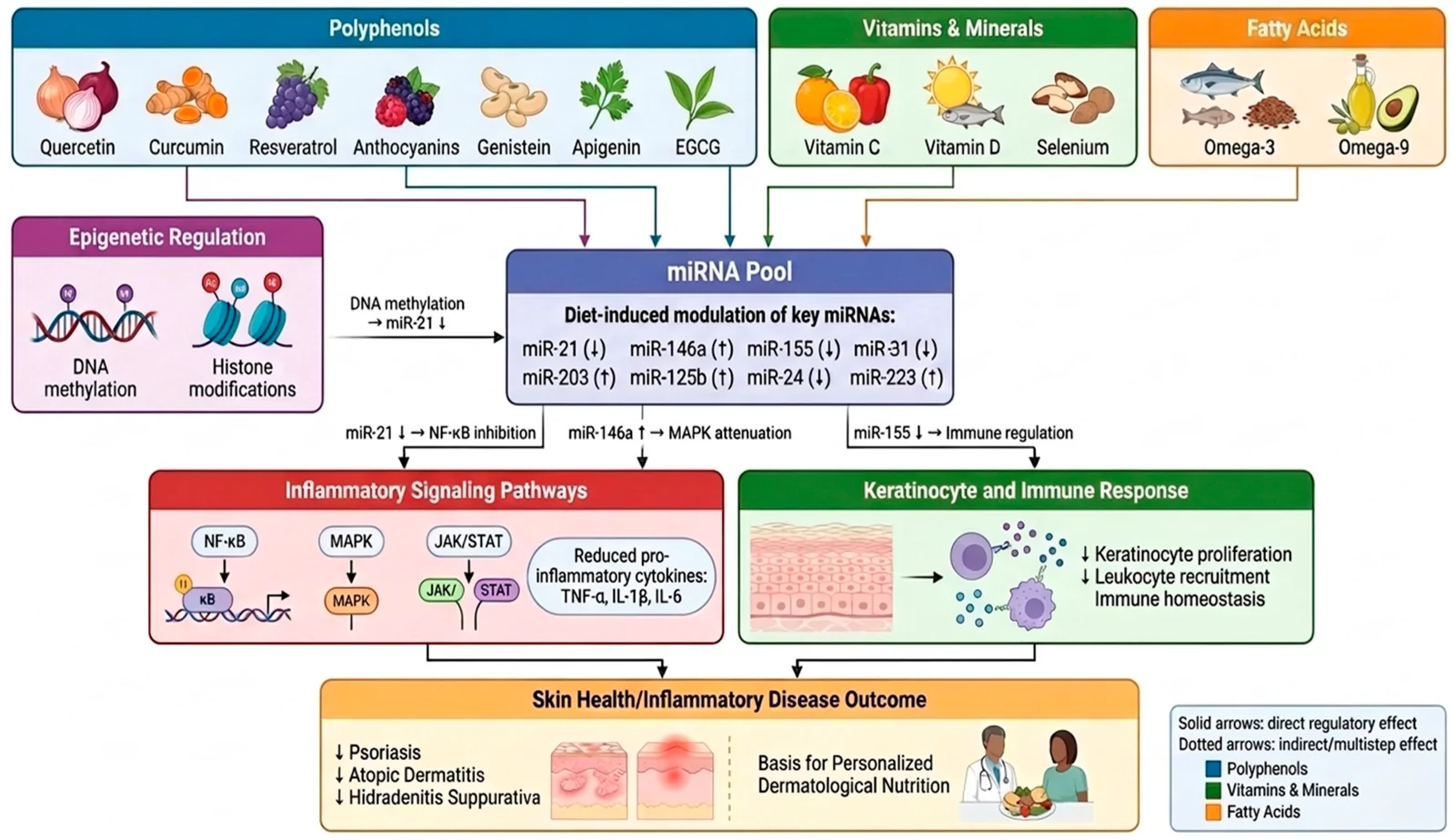
Proposed mechanistic overview of dietary component-driven microRNA modulation in inflammatory skin diseases. Bioactive nutrients (polyphenols, omega-3 fatty acids, vitamins) modulate specific endogenous miRNAs (e.g., miR-21, miR-146a, miR-155) in keratinocytes and immune cells. These microRNA alterations target key intracellular cascades (including NF-κB, MAPK, and JAK/STAT) and may be associated with pro-inflammatory cytokine production (TNF α-, IL-6, IL-17) and the restoration of cutaneous cellular homeostasis. Not all nutrient–miRNA–pathway interactions have been functionally validated, particularly in human dermatologic models; therefore, the relative contribution of miRNA-dependent and miRNA-independent mechanisms remains to be clarified.

Inflammatory and autoimmune skin diseases, such as psoriasis, atopic dermatitis (AD), and hidradenitis suppurativa represent significant therapeutic challenges due to their complex pathogenesis, encompassing immunological, genetic, and environmental factors. In recent years, particular attention has been drawn to the role of epigenetic mechanisms, and specifically microRNA (miRNA) in the regulation of inflammatory processes. MicroRNAs are small, non-coding, single-stranded RNA molecules of 19–25 nucleotides in length, which play a crucial role in post-transcriptional regulation of gene expression [1]. Their mechanism of action involves binding to complementary sequences in messenger RNA (mRNA), leading to inhibition of translation or degradation of the target mRNA. Consequently, alterations in microRNA expression profiles actively contribute to the disruption of homeostatic cellular processes, driving the exacerbation of various pathological states, including skin diseases [2]. Simultaneously, the emerging field of nutrigenomics, which investigates the effects of dietary components on gene expression, has highlighted the role of nutrition in modulating molecular signaling pathways, reinforcing the clinical observation that dietary patterns could influence the course and severity of inflammatory dermatoses. Numerous experimental and early clinical observations suggest that bioactive compounds in food may modulate miRNA expression profiles, offering a novel perspective in alleviating symptoms and providing significant support in the treatment of inflammatory skin diseases [3]. However, a gap remains in current dermatological literature regarding the precise molecular mechanisms connecting metabolic intake and cutaneous epigenetic remodeling. To address this ambiguity, this review explores the core scientific question of whether and how specific dietary components may possibly improve inflammatory skin diseases by actively modulating miRNA expression profiles. The purpose of this paper is to provide a comprehensive, mechanistic overview of the cross-talk between bioactive nutrients and epigenetic regulation in the skin. The scope of this review encompasses a detailed analysis of selected macronutrients, micronutrients, vitamins, and polyphenols, focusing specifically on their ability to regulate key dysregulated miRNAs—including miR-31, miR-21, miR-146a, miR-155, miR-203, miR-125b, miR-24, and miR-223—in the context of psoriasis, AD, and HS. Based on the available literature, this review examines dietary bioactives as potential epigenetic modulators that may influence dysregulated miRNA profiles and may contribute, in part, to the modulation of pro-inflammatory cascades (such as nuclear factor kappa-light-chain-enhancer of activated B cells [NF-κB] and Janus kinase/Signal Transducer and Activator of Transcription [JAK/STAT] pathways). The strength of evidence differs substantially across nutrients, miRNAs, experimental systems, and clinical outcomes; therefore, the proposed nutrient–miRNA–disease relationships should primarily be regarded as biologically plausible and hypothesis-generating. Rather than establishing personalized dietary recommendations for routine dermatological practice, this review aims to outline an emerging framework in which validated nutritional, molecular, and clinical data could eventually contribute to more individualized supportive management of inflammatory skin diseases. Ultimately, this review highlights nutritional–miRNA interactions as an emerging area of translational research [Figure 1].

### Review Methodology

Electronic literature searches were performed in the PubMed/MEDLINE, Scopus, and Web of Science databases. The articles for this review were published up to February 2026. The following search phrases were used: (‘microRNA’ OR ‘miRNA’ OR ‘epigenetics’) AND/OR (‘diet’ OR ‘nutrition’ OR ‘nutrigenomics’ OR ‘polyphenols’ OR ‘fatty acids’ OR ‘vitamins’) AND/OR (‘psoriasis’ OR ‘atopic dermatitis’ OR ‘hidradenitis suppurativa’) as key words or MeSH terms. Inclusion criteria prioritized primary randomized controlled trials, prospective human cohorts, mechanistic in vitro and in vivo studies evaluating cutaneous or cellular miRNA regulation, and recent meta-analyses published in peer-reviewed English-language journals. Duplicates and non-English language articles were excluded. Full-text articles were screened independently by two authors (A.C. and K.D.). Studies lacking clear nutritional or epigenetic endpoints, non-dermatological case reports, and non-peer-reviewed records were excluded. 

## 2. Characteristics of Selected Dietary Components Exhibiting Nutrigenomic Modulatory Action

### 2.1. Polyphenols

Polyphenols are a diverse group of phytochemical compounds characterized by the presence of multiple phenolic groups (-OH) attached to aromatic rings. They constitute one of the most common classes of secondary plant metabolites, serving protective functions against UV radiation, pathogens, and oxidative stress. The biochemical classification encompasses four main groups: flavonoids (quercetin, catechins, and anthocyanins), phenolic acids (gallic acid, caffeic acid, and chlorogenic acid), stilbenes (resveratrol), and lignans. Each class exhibits specific biological properties and varying bioavailability. Recommended daily polyphenol intake is approximately 500–1000 mg. Polyphenols, being natural antioxidants present in fruits, vegetables, green tea, and olive oil, exhibit multidirectional biological action. A diet rich in polyphenols, including the Mediterranean diet, has been associated with changes in the expression of several miRNAs in selected experimental and human studies; the consistency and skin-specific relevance of these observations remain uncertain [4]. An in vivo study conducted by Milenkovich and colleagues demonstrated for the first time that polyphenols, including quercetin, curcumin, ferulic acid, hesperidin, naringin, anthocyanin, catechin, proanthocyanidin, and caffeic acid at nutritional doses modulate miRNA expression in the liver [5].

#### 2.1.1. Quercetin

Quercetin (3,3′,4′,5,7-pentahydroxyflavone) is a bioactive flavonoid occurring in many fruits and vegetables, including onions, capers, apples, grapes, broccoli, and green tea [6]. In experimental and selected clinical contexts, quercetin has been associated with numerous health benefits, including antioxidant, anti-inflammatory, anti-tumor, anti-apoptotic, antihypertensive, and neuroprotective effects [6,7]. These actions are related to quercetin’s ability to modulate canonical biochemical signaling pathways and exert multi-level effects on epigenetic networks. Recent research suggests its role as a natural regulator of DNA methylation, histone modifications, and microRNA expression, with important implications in the prevention of metabolic and neoplastic diseases [7]. In the context of anti-inflammatory action, quercetin significantly inhibits protein regulators such as NF-κB, AP-1, and mitogen-activated protein kinase (MAPK) (extracellular signal regulated kinase [ERK] and c Jun N terminal kinase [JNK]), decreasing the expression of tumor necrosis factor alpha, interleukin (IL)-1, IL-2, IL-6, IL-8, IL-12, cyclooxygenase 2, or prostaglandin E2 [8,9]. These anti-inflammatory effects have been described across different experimental settings and should not be assumed to be uniformly miRNA-dependent. Although quercetin can modulate miRNA expression, the contribution of individual miRNAs to its effects on NF-κB, MAPK, and inflammatory cytokines requires functional validation for each proposed molecular axis. In inflammatory skin diseases, quercetin inhibits keratinocyte hyperproliferation, and tyrosinase activity, and reduces melanin production. It supports wound healing by increasing fibroblast proliferation, epithelialization, and collagen production [9]. However, the absorption and bioavailability of quercetin may prove to be important factors affecting bioeffectiveness [10].

#### 2.1.2. Anthocyanins

Anthocyanins are water-soluble flavonoids responsible for the red, purple, and blue colors of fruits, vegetables, and flowers. Dietary sources of anthocyanins include red and purple berries, grapes, apples, plums, cabbage, and food products containing high amounts of natural pigments. The six dominant anthocyanidins occurring in food are cyanidin, delphinidin, pelargonidin, peonidin, petunidin, and malvidin [11]. Upon consumption, anthocyanins are absorbed in the gastrointestinal tract, with most absorption and metabolic processes occurring in the distal colon. The health benefits of anthocyanins have been extensively described, particularly in the prevention of diseases associated with oxidative stress, such as cardiovascular diseases and neurodegenerative diseases [11,12,13]. The strong antioxidant properties of anthocyanins are achieved through the neutralization of reactive oxygen species (ROS) and the modulation of important signaling pathways, such as nuclear factor erythroid derived 2 (Nrf2) and NF-κB [14]. 

A systematic review and meta-analysis by Hariri et al. investigated the specific impact of purified anthocyanins supplementation on systemic inflammatory mediators across randomized clinical trials [15]. Overall, pooled estimates indicated statistically significant reductions in serum C-reactive protein (CRP: WMD = −0.12 mg/L, 95% CI: −0.21 to −0.02, *p* = 0.013; I^2^ = 76.7%), tumor necrosis factor-alpha (TNF-α: WMD = −1.37 pg/mL, 95% CI: −1.79 to −0.96, *p* < 0.001; I^2^ = 95.3%), and interleukin-6 (IL-6: WMD = −1.43 pg/mL, 95% CI: −1.87 to −1.00, *p* < 0.001; I^2^ = 98.0%) [15]. However, a critical sub-group analysis reveals important boundary conditions and null effects that qualify these broad assertions. Significant reductions in CRP were restricted exclusively to unhealthy/at-risk populations, higher daily dosages (320 mg/day: WMD = −0.18 mg/L, *p* = 0.032), and longer treatment durations (84 days: WMD = −0.18 mg/L, *p* = 0.041) [15]. Assessment of publication bias via Egger’s test revealed significant small-study effect/publication bias for TNF-α (*p* = 0.016) and IL-6 (*p* = 0.001), while no significant bias was detected for CRP (*p* = 0.312) [15]. These findings highlight that while purified anthocyanins may suppress chronic low-grade systemic inflammation, their clinical effectiveness depends heavily on baseline inflammatory burden, adequate dosing, and sustained long-term administration [15]. Importantly, the clinical trials included in this meta-analysis evaluated systemic inflammatory markers rather than miRNA-mediated mechanisms. Therefore, the observed reductions in CRP, TNF-α, and IL-6 should be interpreted as clinical anti-inflammatory effects of anthocyanin supplementation; they do not, by themselves, demonstrate miRNA dependence. Moreover, the effects of anthocyanins on increased levels of anti-inflammatory cytokines, such as IL-10, have also been described [14]. Additionally, recent research suggests that the health-promoting effects of anthocyanins may also be related to modulation of the gut microbiome [11]. 

#### 2.1.3. Resveratrol 

Resveratrol (3,5,4′-trihydroxy-trans-stilbene) is a natural polyphenol from the stilbene group occurring in grape skins, red wine, nuts, and berries. Given its broad range of reported molecular effects, it may be considered a “lifestyle epigenetic modulator”: it exhibits antioxidant, anti-inflammatory, neuroprotective, and cardioprotective properties of nutrigenomic character [16]. It has been demonstrated that resveratrol intake (500 mg daily for 30 days) reduces cardiovascular disease risk factors by increasing sirtuin 1 (SIRT1) levels, improving total antioxidant capacity in healthy individuals, decreasing low-density lipoprotein cholesterol (LDL-C), apolipoprotein B (ApoB), and oxidized LDL levels [16]. Numerous molecular mechanisms modified by resveratrol and contributing to its antioxidant properties have been described in studies. Resveratrol increases the expression of phosphatase and tensin homolog (PTEN), decreasing protein kinase B (Akt) phosphorylation and leading to a subsequent increase in the mRNA levels of antioxidant enzymes, such as catalase (CAT) and superoxide dismutase (SOD). Additionally, it is responsible for promoting antioxidant molecules and the expression of genes associated with mitochondrial energy biogenesis [16]. Resveratrol has been reported to regulate pro- and anti-inflammatory cytokines and chemokines, in part through increased SIRT1 expression, suppression of NF-κB-related signaling, and inhibition of NOD-, LRR- and pyrin domain-containing protein 3 (NLRP3) inflammasome activation. Although resveratrol can also alter the expression of selected miRNAs, the available evidence does not establish that its effects on NF-κB or NLRP3 are universally miRNA-dependent. These pathways may be regulated through both miRNA-related and miRNA-independent mechanisms, depending on the experimental context [16].

#### 2.1.4. Genistein

Genistein belongs to the flavonoid family, a subgroup of isoflavones. It is a phytoestrogen derived mainly from legume plants, such as lupin, broad bean, soybean, pea, common bean, chickpea, and red clover [17]. Numerous biological effects of genistein have been described to date, including antioxidant, anti-inflammatory, antibacterial, and antiviral effects [17,18]. In the anti-inflammatory context, genistein has been reported to modulate various signaling pathways, such as NF-κB, prostaglandins (PG), inducible nitric oxide synthase (iNOS), pro-inflammatory cytokines, and reactive oxygen species (ROS) [18]. 

#### 2.1.5. Curcumin

Curcumin is classified as a polyphenol. It is the main bioactive component of turmeric (*Curcuma longa*) and has demonstrated anti-inflammatory properties and modulating gene expression. Increasing evidence suggests that curcumin exhibits a range of pharmacological properties, including anti-tumor, hepatoprotective, anti-inflammatory, antioxidant, and antibacterial properties [19]. Additionally, curcumin supplementation has been investigated for beneficial modulation of hyperlipidemia, insulin resistance, and glucose tolerance, suggesting potential therapeutic value in alleviating metabolic disorders, such as obesity, diabetes, and cardiovascular diseases [20]. A comprehensive GRADE-assessed systematic review and meta-analysis of 66 RCTs by Dehzad et al. evaluated the systemic anti-inflammatory and antioxidant effects of turmeric and curcumin supplementation in adults [21]. The pooled analysis demonstrated statistically significant reductions in circulating CRP (WMD = −0.58 mg/L, 95% CI: −0.74 to −0.41; I^2^ = 98.9%), TNF-α (WMD = −3.48 pg/mL, 95% CI: −4.38 to −2.58; I^2^ = 99.4%), and interleukin-6 (IL-6: WMD = −1.31 pg/mL, 95% CI: −1.58 to −0.67; I^2^ = 88.2%) [21]. Moreover, curcumin improved systemic antioxidant status by enhancing total antioxidant capacity (TAC: WMD = 0.21 mmol/L, 95% CI: 0.08 to 0.33; I^2^ = 99.6%) and superoxide dismutase activity (SOD: WMD = 20.51 U/L, 95% CI: 7.35 to 33.67; I^2^ = 95.4%), alongside a reduction in malondialdehyde levels (MDA) (MDA: WMD = −0.33 µmol/L, 95% CI: −0.53 to −0.12; I^2^ = 99.6%) [21]. However, curcumin supplementation failed to produce any statistically significant reduction in interleukin-1 beta levels (IL-1β: WMD = −0.46 pg/mL, 95% CI: −1.18 to 0.27, *p* = 0.218; I^2^ = 75.8%) [21]. Evaluation of publication bias via Egger’s test revealed significant asymmetry specifically for IL-1β (*p* = 0.038), though adjustment via the trim-and-fill method confirmed the absence of a true therapeutic effect (adjusted WMD = −0.46 pg/mL, 95% CI: −1.18 to 0.27). Egger’s test did not detect significant publication bias for CRP, TNF-α, IL-6, TAC, MDA, or SOD [21]. The included clinical studies assessed circulating inflammatory and oxidative-stress markers but did not determine whether these changes were mediated by alterations in miRNA expression. Accordingly, the observed reductions in CRP, TNF-α, and IL-6 provide evidence of systemic anti-inflammatory effects of curcumin supplementation, but do not establish a miRNA-dependent mechanism.

#### 2.1.6. Apigenin

Apigenin is one of the most abundant flavonoids occurring in plants with a flavone structure. The best sources of apigenin are parsley, chamomile, celery, spinach, artichokes, and oregano. Among fresh herbs, the highest content of apigenin is found in parsley (1484 mg per 100 g). Apigenin is also a component of red wine and beer [22]. Numerous therapeutic properties of apigenin have been described to date, including antioxidant, anti-tumor, antiviral, anti-inflammatory, antimutagenic, and antibacterial effects. Apigenin has the ability to stimulate the production of antioxidant enzymes, such as glutathione synthetase (GSH), catalase (CAT), and SOD. Additionally, through its effects on cytokines such as (IL)-1β, IL-2, IL-6, IL-8, TNF-α, AP-1 factors, and cyclooxygenase (COX)-2, it exhibits strong anti-inflammatory effects [22]. Apigenin supports various anti-inflammatory pathways, including p38/MAPK and phosphoinositide 3 kinase (PI3K)/Akt, and also prevents inhibitor of kappa B [IKB] degradation and nuclear translocation of NF-κB [23]. In human cell cultures, apigenin demonstrated the ability to inactivate NF-κB by inhibiting phosphorylation of the p65 subunit induced by lipopolysaccharide (LPS). Although apigenin has also been reported to modulate miR-155 expression in experimental models, the available evidence does not establish that its effects on NF-κB or p38 MAPK signaling are mediated by miR-155. In particular, it remains necessary to distinguish inhibition of NF-κB as an upstream regulator of miRNA expression from a miRNA-dependent effect on NF-κB signaling [23]. The role of apigenin has also been investigated in the context of skin effects, where apigenin demonstrated beneficial effects on skin inflammation, wrinkles, UV-induced skin inflammation, vitiligo, wound healing, and skin cancer [22]. 

#### 2.1.7. Epigallocatechin Gallate (EGCG) 

Epigallocatechin gallate (EGCG) is one of the most abundant and most active polyphenols in green tea (*Camellia sinensis*), constituting over 50% of catechin content. It exhibits strong antioxidant, anti-inflammatory, cardioprotective, and anti-tumor properties [24]. It has been demonstrated that EGCG affects various physiological pathways, including lipid metabolism and immune functions, and may even modulate the microbiological balance in the intestines [24]. The phenolic rings in EGCG act as electron traps and scavengers of free radicals, inhibiting the formation of reactive oxygen species (ROS) and mitigating oxidative damage. The antioxidant effects of catechins, including EGCG, are more pronounced than vitamins C and E [24]. Epigallocatechin gallate exhibits strong anti-inflammatory properties through the modulation of key signaling pathways in the cell. Both in vitro and in vivo studies demonstrate that epigallocatechin gallate effectively inhibits the activation of the transcription factor NF-κB by blocking its translocation to the cell nucleus. Additionally, EGCG limits the activity of the activator protein 1 (AP-1) protein, which together results in a reduction in the expression of enzymes responsible for inflammatory processes, such as iNOS and COX-2. This effect also translates into neutralization of reactive oxygen and nitrogen species, including nitric oxide and peroxynitrite, which contribute to the exacerbation of oxidative stress and inflammatory processes [24].

### 2.2. Vitamins and Minerals

Vitamin D is essential for the functioning of almost every tissue, including the cardiovascular system, bones, immune system, and skin. The active form is the 1,25-hydroxylated form [25]. Deficiency may occur due to a diet with insufficient vitamin D content, impaired absorption, and limited sun exposure. Products rich in vitamin D include fish, liver, margarine, milk, eggs, and meat [25]. Vitamin D affects the differentiation and maturation of immune system cells. By inhibiting NF-κB and mitogen-activated protein kinase (MAPK) pathways, vitamin D reduces the expression of pro-inflammatory genes, such as TNF-α, IL-1β, IL-6, and IL-8. These anti-inflammatory effects of vitamin D have been described independently of miRNA assessment in many experimental and clinical studies [25]. Simultaneously, vitamin D stimulates the synthesis of anti-inflammatory cytokines, such as interleukin-10 (IL-10) [25]. Results of studies on the anti-inflammatory properties of vitamin D suggest that it may play an important therapeutic role in the treatment of inflammatory diseases [25]. Additionally, through inducing the expression of such molecules as glutathione, glutathione peroxidase, and superoxide dismutase and inhibiting the expression of nicotinamide adenine dinucleotide phosphate (reduced form) (NADPH) oxidase, vitamin D exhibits antioxidant effects [25].

Vitamin C (ascorbic acid) is a water-soluble antioxidant essential for the proper functioning of the human body. It serves as a cofactor for hydroxylases responsible for collagen synthesis, which affects wound healing and skin elasticity, and facilitates iron absorption by reducing Fe^3+^ to Fe^2+^. It supports immune mechanisms by increasing interferon secretion and phagocyte activity, while simultaneously neutralizing free radicals, protecting cells from oxidative stress [26]. The best sources of vitamin C include wild rose berries, blackcurrants, citrus fruits, and vegetables such as peppers, broccoli, cauliflower, and parsley [27]. In a study by Ellulu et al. investigating the effects of vitamin C on inflammatory markers (IL-6 and CRP levels in serum) and metabolic markers in adults with hypertension and/or diabetes and obesity, the experimental group was given 500 mg of vitamin C twice daily. It was demonstrated that vitamin C supplementation significantly reduced the level of high-sensitivity C-reactive protein (hs-CRP), interleukin 6 (IL-6), fasting blood glucose (FBG), and triglycerides (TG) after 8 weeks of treatment. As miRNA expression and functional miRNA–target interactions were not assessed in that study, the reductions in hs-CRP and IL-6 cannot be attributed to a miRNA-dependent mechanism [28]. In cross-sectional studies conducted by Wannamethee et al., assessing the relationship between inflammatory markers and blood vitamin C concentrations, it was found that plasma vitamin C concentration correlated with a significant reduction in the risk of heart failure in men with previously occurring myocardial infarction or without it [29]. At the nutrigenomic level, vitamin C affects the expression of genes involved in inflammatory and immunological processes. Research from recent years has demonstrated that vitamin C supplementation in healthy individuals modulated gene expression following exposure to an inflammatory stimulus (LPS), including elements of the myeloid differentiation primary response 88 (MyD88) pathway and IL-10 synthesis [30].

Vitamin E is a group of fat-soluble compounds, with α-tocopherol being the biologically most active and best-studied form, characterized chemically by a chromanol ring and a hydrophobic phytyl side chain [31]. It is a lipophilic antioxidant whose primary function is to protect cell membranes against lipid peroxidation and oxidative damage. In dermatology, this is particularly relevant because oxidative stress contributes to the pathogenesis of many inflammatory skin diseases, including atopic dermatitis and psoriasis [32]. 

A meta-analysis performed by Liu et al. indicated that serum vitamin E levels were lower in patients suffering from vitiligo, psoriasis, atopic dermatitis and acne [33]. In patients with psoriasis, it was additionally observed that those with the most severe disease had the lowest vitamin E levels [34]. Available clinical data suggest that vitamin E supplementation may be beneficial in selected inflammatory skin diseases, although the quality of evidence remains limited and does not allow for definitive conclusions. 

Vitamin E supplementation has been investigated in atopic dermatitis, with mixed findings regarding its potential effect on disease activity. In the study by Tsoureli-Nikita et al., 96 patients with AD were randomly assigned to two groups: one receiving natural vitamin E at a dose of 400 IU/day and the other receiving a placebo for 8 months. By the end of the study, nearly complete remission of AD was observed in seven participants receiving daily vitamin E, whereas no such remission occurred in the placebo group. Overall, marked improvement in AD was seen in 23 of 50 subjects treated with vitamin E, compared with only one subject in the placebo group [34]. Similar findings were reported in the study by Jaffary et al. It was a randomized, double-blind, placebo-controlled study evaluating vitamin E supplementation in 70 patients with mild-to-moderate AD. Participants were assigned to receive either 400 IU of vitamin E daily (*n* = 35) or placebo (*n* = 35). After 4 months, the vitamin E group demonstrated less pruritus, a lower SCORAD score, and smaller lesions than the placebo group, with statistically significant differences in itching, lesion extent, and overall SCORAD index (*p* < 0.05) [35]. On the other hand, the results published in 1989 by Fairris et al. on selenium and vitamin E supplementation at a daily dose of 600 IU/day in patients with AD did not confirm any association between supplementation and a reduction in disease activity, as assessed after 12 weeks of treatment compared with placebo [36]. In psoriasis, Kvaraeva et al. evaluated the impact of an antioxidant mix composed of coenzyme Q10 (ubiquinone acetate, 50 mg/day), vitamin E (natural alpha-tocopherol, 50 mg/day), and selenium (aspartate salt, 48 mg/day) in patients with severe disease. In a randomized, double-blind trial, 28 individuals with severe psoriatic arthritis and 30 with erythrodermic psoriasis were assigned either to conventional therapy plus the antioxidant mix or to conventional therapy plus placebo. Over the course of the study, clinical responses occurred more rapidly in the supplemented groups than in the controls, and by day 30 disease severity, based on subjective scoring, was significantly lower in patients receiving antioxidants in addition to standard treatment [37].

Coenzyme Q10 (CoQ10, ubiquinone) is a fat-soluble, vitamin-like molecule present in all cell membranes. It is a key component of the mitochondrial respiratory chain, where it participates in energy production (ATP). CoQ10 also plays an important role as an antioxidant, protecting cells from oxidative stress and supporting the regeneration of other antioxidants, such as vitamin E [38]. CoQ10 supplementation significantly reduces inflammatory markers: CRP, interleukin-6 (IL-6), and TNF-α [32]. These findings reflect systemic anti-inflammatory effects reported outside dermatologic disease and do not provide direct evidence that CoQ10 modifies miRNA-related inflammatory mechanisms in the skin. The most commonly used doses are 100–400 mg per day, and higher doses may be beneficial in reducing inflammatory markers [39].

Lycopene is a natural carotenoid with strong antioxidant properties, constituting the red pigment occurring primarily in tomatoes, watermelon, grapefruit, and guava. Recent research suggests its multidirectional health-promoting effects, particularly in the prevention of cancer, cardiovascular, and metabolic diseases [40,41,42,43]. It contributes to reducing the expression of interleukins, such as IL-1beta, IL-6, and TNF-α, and affects the neutralization of oxidative stress, thereby preventing DNA damage caused by reactive oxygen species (ROS) [41]. 

Selenium is an essential micronutrient acting as a cofactor for selenoproteins, including glutathione peroxidase (GPx), thioredoxin reductase (TrxR), and selenoprotein P, which play key antioxidant (neutralizing reactive oxygen and nitrogen species), detoxification, anti-inflammatory, immunomodulatory, and anti-tumor functions [44]. Selenium has minimal impact on inflammatory state initiation but effectively regulates its progression [45]. A systematic review of 76 meta-analyses showed that dietary selenium intake at the highest dose may reduce the risk of death from any cause in adults compared to the lowest dose. Dietary selenium intake in monotherapy may correlate with reduced risk of developing metabolic syndrome and depression, while in combination with specific medications it may improve symptoms and some disease markers of several conditions, including Graves-Basedow disease, polycystic ovary syndrome, and cardiovascular diseases, compared to placebo [46].

### 2.3. Fatty Acids 

Omega-3 fatty acids are a group of polyunsaturated fatty acids essential for human health, comprising alpha-linolenic acid (ALA), eicosapentaenoic acid (EPA) and docosahexaenoic acid (DHA). ALA is mainly derived from plant sources (e.g., flaxseed, flaxseed oil, and walnuts), whereas EPA and DHA are found predominantly in fatty marine fish and seafood. Beyond their structural role as components of cell membranes, these fatty acids are precursors of a wide range of bioactive lipid mediators that critically regulate inflammatory responses, including in the skin. EPA and DHA can compete with arachidonic acid (AA, n-6 polyunsaturated fatty acids [PUFA]) for cyclooxygenase and lipoxygenase pathways, thereby modulating the production of prostaglandins and leukotrienes and shifting the balance from pro-inflammatory AA-derived mediators (e.g., prostaglandin E_2_ [PGE_2_], and leukotriene B4 [LTB_4_]) towards less potent or even pro-resolving n-3–derived metabolites [47,48,49].

From EPA and DHA, specialized pro-resolving mediators (SPMs) such as resolvins, protectins and maresins are generated, which actively terminate inflammation, promote clearance of neutrophils and restore tissue homeostasis. Experimental and clinical data indicate that omega-3 PUFAs exert anti-inflammatory and immunomodulatory effects in inflammatory diseases, partly by reducing pro-inflammatory cytokines and eicosanoids and enhancing the formation of SPMs. In inflammatory skin disorders, including psoriasis and atopic dermatitis, disturbances in PUFA-derived lipid mediator pathways with a predominance of omega-6-driven prostanoids and leukotrienes and reduced omega-3-derived SPMs have been linked to a persistent, non-resolving inflammatory milieu in lesional and non-lesional skin. Consequently, dietary omega-3 fatty acids should be considered not only as nutritional factors but also as modulators of cutaneous immune responses through their impact on the prostaglandin, leukotriene and SPM networks relevant to inflammatory skin diseases [47,50,51]. 

Oleic acid is a monounsaturated omega-9 fatty acid (MUFA), occurring widely in plant and animal fats, particularly in olive oil, rapeseed oil, almond oil, avocado, as well as in fish oil and egg yolk [47]. In the diet, oleic acid favorably influences serum lipid profiles and blood pressure and can partially replace dietary saturated fats, thereby exerting cardiometabolic benefits. In the skin, oleic acid is incorporated into membrane phospholipids and surface lipids, contributing to barrier function and modulating the fluidity and organization of the stratum corneum, which may indirectly affect the generation and action of eicosanoids and other lipid mediators involved in cutaneous inflammation. Topically, it is widely used in cosmetic and dermatologic formulations for its emollient, regenerative and skin-softening properties, and in the context of inflammatory skin diseases, omega-3 PUFAs and omega-9 MUFAs together represent key dietary determinants of the cutaneous lipid mediator milieu, with potential to shift the balance between pro-inflammatory prostaglandins/leukotrienes and pro-resolving mediators [47].

## 3. Nutrigenetic Mechanisms Modulating Gene Expression and Cell Function

A growing body of evidence indicates the possibility of modulating the expression of endogenous microRNAs through dietary factors. Nevertheless, altered miRNA expression should not be equated with a miRNA-dependent biological effect. Evidence for miRNA dependence requires functional validation, ideally through miRNA gain- or loss-of-function approaches, confirmation of relevant mRNA targets, and demonstration that manipulation of the miRNA modifies the effect of the dietary compound on the downstream pathway or phenotype. A key element of nutrigenomics is understanding how bioactive compounds contained in food can modulate epigenetic pathways. One of the main types of modulation is DNA methylation, A diet rich in methyl donors (such as folic acid, vitamin B12, choline, and methionine) can influence methylation levels through one-carbon metabolism. Nutrients participating in one-carbon metabolism regulate the delivery of methyl groups for biological methylation reactions, including DNA and histone proteins [52,53]. Another mechanism of nutrigenomics is histone modification. Bioactive substances, such as polyphenols, can modulate enzymes that modify histones (histone deacetylases [HDACs], histone acetyltransferases [HATs]), which changes the activity of genes associated with inflammatory processes and skin regeneration. Reactive oxygen species change nuclear acetylation and deacetylation of histones, leading to increased expression of pro-inflammatory mediator genes dependent on NF-κB [54,55]. Regulation involving the binding of non-coding micro-RNA to mRNA followed by degradation of the target molecule or blockade of translation is another mechanism observed in nutrigenomics. Dietary components can change the levels and expression profiles of miRNA, which directly affects the regulation of pro-inflammatory, immunomodulatory genes, and those involved in skin homeostasis [56,57]. A systematic review of 37 studies showed that energy- and fat-controlled diets and plant-based foods show significant evidence for modulating levels of endogenous miRNA [57]. A total of 108 miRNAs modulated by diet were identified, with energy content and fat intake proving to be key factors influencing miRNA levels [57]. Particularly interesting are studies on the effects of the Mediterranean diet on microRNA expression. This dietary model, characterized by high consumption of vegetables, fruits, olive oil, fish, and nuts, and low consumption of red meat and processed products, exhibits strong anti-inflammatory properties. Some components of this diet, such as polyphenols, polyunsaturated omega-3 fatty acids, and dietary fiber, can influence the expression of miRNAs associated with the regulation of inflammatory processes [4].

Nutrigenomic modification may also include effects on transcription factors and signaling pathways—some dietary components activate or inhibit transcription factors, such as NF-κB, Nrf2, STAT, which modulate inflammatory responses and oxidative stress. The transcription factor Nrf2 in skin cells is responsible for the transcription of genes encoding antioxidant proteins and plays a crucial role in the protection against oxidative modifications. Natural substances such as curcumin, sulforaphane, and broccoli leaf extracts cause selective activation of Nrf2 [58,59].

Another example of modification in nutrigenomics is the effect on gut metabolism and the microbiome. Dietary components modulate the composition and functioning of the gut microbiota, which subsequently influences the immune system and epigenetic mechanisms regulating skin gene expression through metabolites. Among nutrients obtained from food, polyunsaturated omega-3 fatty acids have diverse beneficial effects on inflammatory diseases, and omega-3 PUFA metabolites exhibit diverse effects on immune and epithelial cells [49]. Polyunsaturated omega-3 fatty acids, primarily EPA and DHA, affect the gut microbiome in three main ways: by modulating the type and abundance of gut microorganisms, changing levels of pro-inflammatory mediators, and regulating levels of short-chain fatty acids [49]. These mechanisms may indirectly influence the expression of microRNAs associated with the regulation of skin inflammatory conditions through the gut-skin axis. Additionally, short-chain fatty acids inhibit the activity of histone deacetylases (HDACs), causing chromatin changes associated with increased expression of target genes and emerging as potential factors counteracting epigenetic changes induced by dysbiosis, particularly targeting metabolic and inflammatory genes through DNA methylation, histone acetylation, microRNA, and long non-coding RNA [60].

Current evidence indicates that diet can also modulate the level of exogenous miRNA in the intestinal lumen. Dietary microRNAs (xenomiRs) derived from plant, animal, and dairy products exhibit surprising stability in the gastrointestinal tract due to the protective effects of extracellular vesicles and carrier proteins. A key element of their absorption is the SID1 transmembrane family member 1 (SIDT1) receptor located in gastric pit cells, which enables transport of exogenous miRNAs under low stomach pH conditions [61]. Animal model studies confirmed that SIDT1-deficient mice showed significantly reduced absorption of dietary miRNA, indicating the crucial role of this receptor in the absorption process. Additionally, intestinal barrier permeability modulated by the gut microbiota and dietary factors may influence the efficiency of this process [62].

Assimilated xenomiRs may serve as regulators of inter-species epigenetic communication. In in vivo studies, oral administration of plant-derived miR-2911 from *Verbascum* inhibited replication of influenza A virus and SARS-CoV-2, and this effect was abolished in SIDT1-deficient mice [61]. Similarly, miRNAs derived from soy (miR-159) demonstrated the ability to inhibit the expression of the transcription factor 7 (TCF7) oncogene in breast cancer cells, reducing cancer cell proliferation [63]. These mechanisms suggest that dietary miRNAs can modulate immunological and metabolic pathways through direct interaction with target mRNAs. Despite these compelling preclinical findings, caution is warranted regarding their direct clinical translation. Current evidence remains largely derived from in vivo animal models and in vitro studies, which may not fully recapitulate the complexity of human physiological responses. Significant hurdles, including the rapid degradation of miRNAs in the gastrointestinal tract, limited systemic bioavailability, and the influence of inter-individual variations in gut microbiome composition, currently preclude the widespread clinical application of these dietary interventions.

Despite promising mechanistic data, the question of the actual bioavailability of dietary miRNAs remains a subject of scientific debate. Some studies do not confirm the detectability of plant miRNAs in systemic circulation following dietary interventions, which may result from limitations of the detection methods (e.g., low sensitivity of qPCR) or rapid degradation of these molecules [63]. Additionally, standardization of research protocols concerning miRNA isolation, result normalization, and validation of analytical methods represents an important challenge for future research.

## 4. Dietary Considerations in Inflammatory Skin Diseases: Current Evidence and Emerging Nutrigenomic Perspectives

### 4.1. Psoriasis

Dietary interventions may be clinically relevant in selected patients with plaque psoriasis, particularly when they address overweight or obesity, metabolic comorbidity, and overall dietary quality. Their dermatological benefit is variable, and they should be regarded as supportive measures rather than substitutes for standard psoriasis therapy. In patients with excess body weight, weight loss of at least 5% is associated with a significant reduction in the Psoriasis Area and Severity Index (PASI) score, as confirmed by randomized trials and systematic literature reviews [64]. Optimization of dietary therapy requires an individualized approach, considering the patient’s metabolic status, the presence of comorbidities, and dietary preferences. The Mediterranean diet model leads to reduction in pro-inflammatory markers, such as TNF-α and IL-6, and promotes an improvement in skin symptoms through modulation of lipid and immunological profiles [64,65]. Limiting highly processed foods, excess alcohol intake, and dietary patterns associated with adverse cardiometabolic profiles may be reasonable within general health-oriented counselling. Simultaneous reduction in of the omega-6 to omega-3 fatty acid ratio to values between 1:1 and 1:5 additionally strengthens the anti-inflammatory effects of the diet [64,66]. Increased intake of dietary fiber and antioxidants has beneficial effects on the gut microbiota, which translates into modulation of immune responses in the skin. Products rich in lycopene, carotenoids, and flavonoids not only neutralize free radicals but also inhibit the expression of pro-inflammatory cytokines, contributing to a reduction in the intensity of psoriatic lesions [67]. Supplementation with EPA and DHA has been evaluated in several clinical contexts. A meta-analysis of randomized controlled trials by Clark et al. indicated that omega-3 PUFA monotherapy significantly improved certain clinical parameters in psoriatic patients, including the PASI score (WMD = −1.58, 95% CI: −2.24 to −0.92; I^2^ = 0%), erythema (WMD = −1.66, 95% CI: −2.52 to −0.81; I^2^ = 99%), and scaling (WMD = −0.69, 95% CI: −1.26 to −0.13; I^2^ = 98%). Subgroup analyses indicated that higher doses (≥1800 mg/day) and shorter intervention durations (≤8 weeks) were associated with greater clinical improvements. However, a critical evaluation reveals significant variability and non-significant effects across other key disease metrics. Specifically, omega-3 supplementation failed to yield statistically significant reductions in pruritus (WMD = 0.25, 95% CI: −0.65 to 1.15; I^2^ = 94%), affected body surface area (%TBSA: WMD = −7.40, 95% CI: −16.75 to 1.95; I^2^ = 99%), desquamation (WMD = −0.33, 95% CI: −1.35 to 0.69; I^2^ = 97%), or plaque infiltration (WMD = −0.31, 95% CI: −0.82 to 0.20; I^2^ = 97%). The substantial heterogeneity observed across most secondary endpoints (I^2^ > 90%) reflects inconsistencies in study designs, varying control substances (e.g., olive oil, paraffin), and diverse baseline disease severities. Assessment of publication bias for erythema using Begg’s (*p* = 0.17) and Egger’s tests (*p* = 0.06) did not show significant bias [67]. Overall, while omega-3 PUFAs offer modest benefits for specific plaque characteristics, current meta-analytic evidence does not support their efficacy as a universal or standalone intervention for overall psoriasis control.

Moreover, support with probiotics and prebiotics may change the composition of the gut microbiota composition, leading to a reduction in the systemic inflammatory status and improvement in dermatological parameters [64,65]. Vitamin D, which plays a role as a regulator of skin immune responses, should be supplemented in cases of confirmed deficiency should be supplemented (400–2000 IU/day). Calciferol, by modulating gene expression in the NF-κB and IL-17 pathways, supports the control of inflammatory processes; however, in populations with appropriate 25(OH)D levels, routine supplementation solely for psoriasis treatment is not recommended [66].

### 4.2. Atopic Dermatitis (AD) 

Implementation of an elimination diet—consisting of the exclusion of potential allergens (cow’s milk, eggs, nuts, soy)—shows minimal benefits in alleviating atopic dermatitis symptoms. The efficacy and safety of this strategy were thoroughly evaluated in a meta-analysis of 10 RCTs (*N* = 599) by Oykhman et al. [68]. Low-certainty evidence demonstrated that dietary elimination yields only a marginal, clinically questionable reduction in eczema severity, with 50% of participants on elimination diets achieving the minimal important difference (MID of 8.7 points in SCORing Atopic Dermatitis [SCORAD]) compared to 41% in unrestricted controls (risk difference [RD] = 9%, 95% CI: 0% to 17%; pooled SCORAD MD = −1.98, 95% CI: −3.78 to −0.09; I^2^ = 34%). Furthermore, dietary exclusion failed to achieve statistically significant improvements in daytime pruritus (MD = −0.21, 95% CI: −0.57 to 0.15; I^2^ = 76%) [68]. However, the level of evidence was assessed as low, and the risk of developing a food allergy as a result of elimination requires attention. 

Increasingly greater support is gained by probiotic and prebiotic supplementation. In an EAACI Task Force meta-analysis of 20 RCTs (*N* = 1387) in non-food-allergic children with AD, Vassilopoulou et al. evaluated gut microbiota modulation via probiotics, synbiotics, and postbiotics [69]. Although the overall analysis showed a statistically significant reduction in SCORAD scores (MD = 5.54, 95% CI: 2.75 to 8.33; I^2^ = 86%), this effect fell short of the minimal clinically important difference (MCID = 8.7 points), indicating that probiotics offer limited standalone clinical benefit. Crucially, strain-specific analyses demonstrated clear null results: *Lactobacillus rhamnosus* supplementation was ineffective compared to placebo (MD = 4.1, 95% CI: −1.1 to 9.2; I^2^ = 88%), postbiotics provided no added advantage over live strains, and higher daily doses (>1 × 10^10^ CFU) did not improve outcomes (MD = 3.53). Significant publication bias was also detected (Egger’s test *p* < 0.001), suggesting an overrepresentation of positive findings in the literature [69]. Overall, while probiotics remain safe, current evidence does not support using single strains like *L. rhamnosus* as primary disease-modifying therapies in pediatric AD [69]. Complementing these pediatric findings, a broader meta-analysis of 25 RCTs (I = 1382) across children and adults by Kim et al. evaluated probiotic supplementation across distinct disease severities, treatment durations, and geographical regions [70]. While the overall effect showed a reduction in SCORAD (SMD = −4.0, 95% CI: −7.3 to −0.7; I^2^ = 97%), detailed subgroup breakdown highlighted clear therapeutic boundaries and non-significant interventions. Probiotic supplementation was entirely ineffective in patients with severe baseline AD (SMD = −3.3, 95% CI: −7.4 to 0.93, *p* = 0.13; I^2^ = 96.9%), demonstrating significant efficacy only in mild-to-moderate disease (SMD = −1.4, 95% CI: −2.2 to −0.7). Similarly, short-term interventions (<3 months) yielded null therapeutic effects (SMD = -2.5, 95% CI: −7.2 to 2.1, *p* = 0.28; I^2^ = 96.0%), whereas prolonged administration (≥3 months) was required to observe benefit (SMD = −5.1, 95% CI: −9.7 to −0.4). Egger’s test confirmed significant publication bias (*p* < 0.01), indicating that while high between-study heterogeneity (I^2^ = 97%) is present, probiotics remain an option as an adjunctive AD therapy [70]. Regarding vitamin D, evidence from observational and interventional studies has been synthesized in recent meta-analyses [71,72]. 

A meta-analysis of 20 studies by Ng and Yew demonstrated that patients with AD had significantly lower serum 25-hydroxyvitamin D [25(OH)D] levels compared with healthy controls (WMD = −7.42 ng/mL, 95% CI: −11.91 to −2.93, *p* < 0.001; I^2^ = 98.9%), with lower concentrations correlating with disease severity when comparing severe versus mild AD (WMD = −7.99 ng/mL, 95% CI: −11.22 to −4.75, *p* < 0.001; I^2^ = 82.3%) and severe versus moderate AD (WMD = −2.95 ng/mL, 95% CI: −5.16 to −0.74, *p* < 0.001; I^2^ = 85.6%) [72]. Furthermore, an updated systematic review and meta-analysis of 11 randomized controlled trials (686 participants) by Nielsen et al. confirmed that oral vitamin D supplementation significantly reduced overall AD severity compared to control groups (SMD = −0.41, 95% CI: −0.67 to −0.16, *p* < 0.01; I^2^ = 58%) [71]. However, a critical evaluation reveals substantial limitations, non-significant (null) sub-group findings, and potential confounding that modify these therapeutic claims. In the observational synthesis by Ng and Yew, the inverse association between 25(OH)D levels and disease severity lost statistical significance in subgroup analyses restricted to higher latitudes (>35°) and high-quality studies (NOS ≥ 6) [72]. In the meta-analysis by Nielsen et al., individual trial outcomes were highly inconsistent, with only 27% (3/11) of included RCTs reporting statistically significant symptom improvements on their own [71]. Subgroup analyses further demonstrated null effects in key clinical scenarios: vitamin D supplementation failed to yield significant benefits in patients with severe baseline AD (SMD = −0.17, 95% CI: −0.55 to 0.21, *p* = 0.19; I^2^ = 42%), during interventions lasting ≥3 months (SMD = −0.14, 95% CI: −0.47 to 0.19, *p* = 0.40; I2 = 0%), or at daily doses ≤1000 IU/day (SMD = −0.41, 95% CI: −1.02 to 0.20, *p* = 0.24; I^2^ = 30%) [71]. Egger’s test did not indicate significant publication bias (*p* > 0.05), though certainty was downgraded to ‘moderate’ via GRADE criteria due to risk of bias and imprecision [71]. However, in a randomized controlled trial in children with severe AD, the addition of 1600 IU of vitamin D daily for 12 weeks increased the reduction in the EASI index value compared to placebo (56.4% vs. 42.1%; *p* = 0.039) [73]. Routine vitamin D supplementation should not be considered a primary universal therapy for AD, but rather an adjunctive strategy tailored to patients with documented baseline deficiency or mild-to-moderate seasonal flares [71,72,73].

Supplementation with omega-3 and omega-6 fatty acids does not show clear benefits in AD prevention or treatment. A literature review from recent years does not confirm the effects of these fatty acids on improving disease progression [74]. Overall dietary patterns, such as the Mediterranean diet, abundant in fruits, vegetables, whole grain products, nuts, and fish, may contribute to lowering inflammatory markers through increased intake of antioxidants and fiber and modulation of the gut microbiota. Although data is limited, high adherence to this pattern is associated with milder AD progression [74,75].

### 4.3. Hidradenitis Suppurativa (HS)

Weight loss in overweight or obese patients represents a key element of hidradenitis suppurativa (HS) control. In a prospective pilot study, application of a 28-day active phase of a very low-calorie ketogenic diet (VLCKD) led to a significant reduction in the Sartorius scale score (Δ%: −24.4 ± 16.6) and in inflammatory markers, such as trimethylamine N-oxide (TMAO), derivatives of reactive oxygen metabolites (dROMs), and oxLDL (*p* < 0.001) [76]. Maintenance of weight loss ≥5% is associated with clinical improvement in HS and a reduction in inflammatory nodules and sinus tracts. Regular adherence to the Mediterranean diet correlates with lower International Hidradenitis Suppurativa Severity Score System (IHS4) scores and Hurley scores [77]. In cross-sectional studies, patients with HS showed significantly lower adherence to this pattern (lower MEDAS scores) and higher consumption of products with a high glycemic index (GI) compared to the control group (*p* < 0.05) [77]. A narrative review meta-analysis emphasizes that the Mediterranean diet may constitute an economical and safe complement to conventional HS therapy [78,79]. A high GI diet and excessive consumption of high-fat dairy products may intensify HS progression. These mechanisms are related to insulin resistance and increased production of pro-inflammatory cytokines. Elimination of excess simple sugars, alcohol, highly processed products, and a reduction in the omega-6 to omega-3 ratio promotes a reduction in NF-κB activity and the production of TNF-α and IL-6 [78]. Current evidence suggests benefits from a diet eliminating brewer’s yeast, milk, and nightshade products (Solanaceae), although they require further randomized trials are required [78]. Zinc and vitamin D supplementation may support the control of inflammatory status, particularly in individuals with documented deficiency in these micronutrients [78]. Additionally, probiotics and prebiotics, through modulation of the gut-skin axis, appear promising in limiting systemic inflammation; however, there is still a lack of sufficient clinical trials confirming their efficacy in HS [78,79].

## 5. Mechanisms of microRNA Action in Inflammatory Skin Diseases and Their Potential Nutrigenomic Modifications

In inflammatory skin diseases, available studies have reported alterations in microRNA expression profiles that may be associated with disease-related inflammatory and epithelial processes. However, these findings remain emerging, and their reproducibility across cohorts, tissue specificity, and value for clinical decision-making require further validation [Table 1]. Distinct miRNA profiles have been identified in inflammatory dermatoses, and their dysregulation causes impaired control of biological pathways, contributing to pathogenic states [80].

### 5.1. miR-31

miR-31 exhibits significant overexpression in keratinocytes of psoriatic lesions, the blood serum of psoriasis patients, and skin lesions in hidradenitis suppurativa. It plays a crucial role in psoriasis pathogenesis by regulating inflammatory pathways and keratinocyte proliferation; in HS, its overexpression suggests its involvement in the inflammatory process, although further research is warranted. The mechanism of action of miR-31 primarily involves the hibition of serine/threonine protein kinase 40 (*STK40*) expression, a negative regulator of the NF-κB pathway, leading to the activation of pro-inflammatory cytokines (including IL-1β). Additionally, by inhibiting the effects on the catalytic subunit of protein phosphatase 6 (*PPP6C*), it disrupts cell cycle regulation and induces keratinocyte hyperproliferation. miR-31 expression is stimulated by transforming growth factor beta 1 (TGF-β1) and pro-inflammatory cytokines (IL-6, IL-22, TNF-α), creating a positive feedback loop and leading to increased leukocyte chemoattraction, keratinocyte hyperproliferation, and endothelial cell activation [2,3].

In a study published by Fereirã et al. examining miRNA expression in patients with diagnosed squamous cell carcinoma of the head and neck, miR-31 expression showed a positive correlation with iron intake (β = 16.65) and vitamin C intake (β = 0.37), and an inverse correlation with total sugar consumption (β = −0.88), cholesterol (β = −0.23), vitamin B9 (β = −0.37), and zinc (β = −5.66) [81]. These findings are based on a cancer model and therefore require validation in skin-specific models to determine their relevance to cutaneous biology.

In turn, in the Desgagne et al. study, the effects of trans fatty acid (TFA) versus un-saturated fatty acid (UFA) consumption on miRNA profiles in high density lipoprotein (HDL) were assessed. Participants consumed meals rich in TFA or UFA, after which changes in plasma and HDL miRNA were analyzed. It was found that miR-31-5p is one of the key microRNAs modulated by TFA, with diets high in trans fats (iTFA and rTFA) significantly reducing its expression in HDL. The observed reduction correlated with impaired cardioprotective functions of HDL [82]. As these findings derive from a circulating HDL/miRNA model rather than a skin model, validation in dermal experimental systems is required.

### 5.2. miR-21 

miR-21 is a pleiotropic regulator of cell biology with the ability to stimulate proliferation, migration, invasion, and the inflammatory cascade, and to inhibit apoptosis [2]. It is one of the most important microRNAs in the pathogenesis of dermatological diseases. miR-21 expression is increased in epidermal lesions in psoriasis patients, leading to a reduction in epidermal tissue inhibitor of matrix metalloproteinase 3 (TIMP-3) expression (tissue inhibitor of matrix metalloproteinase 3) and activation of tumor necrosis factor-α-converting enzyme TACE/ADAM17 (a disintegrin and metallopro-teinase 17) [83]. Overexpression of miR-21 has also been found in tissue samples and serum of patients with atopic dermatitis compared to the control group [84]. miR-21 also plays a role in the etiopathogenesis of acne vulgaris, where its effects are related to mechanisms of oxidative stress. Calis et al. also demonstrated that miR-21 levels in plasma were statistically significantly higher in patients with severe acne compared to the control group [(0.55 ± 0.06) vs. (0.30 ± 0.05), *p* = 0.003]. Based on these findings, the researchers suggested that regulation of miR-21 and the use of antioxidants in combination with various acne treatment methods may be beneficial, especially in cases of severe acne [85]. miR-21 levels also showed significant correlations with several autoimmune diseases, including systemic lupus erythematosus (SLE). In patients with active SLE, this molecule was found to be overexpressed in serum samples and positively correlated with the systemic lupus erythematosus disease activity index 2K (SLE-DAI Index2K) and activity of the SLE disease activity score 2K (SLE-DAI 2K activity) [86]. In contrast, in the case of vitiligo, miR-21 shows decreased expression in peripheral blood mononuclear cells and may play a protective role in its pathogenesis, affecting the balance between Treg/Teff in the CD4+ cell population [87]. Beyond inflammatory dermatoses, miR-21 also acts as an oncogene, exhibiting increased expression during progression from benign melanocytic lesions to malignant melanoma, which is associated with the presence of v Raf murine sarcoma viral oncogene homolog B (BRAF) and neuroblastoma RAS viral oncogene homolog (NRAS) mutations [88]. Current evidence from the literature unequivocally indicates that dietary components and lifestyle affect miR-21 expression. Some of these components are polyphenols, such as curcumin, resveratrol, EGCG, quercetin, and genistein. 

Curcumin exhibits modulatory properties toward miR-21. Studies demonstrated that curcumin inhibits miR-21 expression in a dose-dependent manner through inhibition of the binding of transcription factor AP-1 to the miR-21 promoter in colorectal cancer [89]. Clinical and in vitro studies in peripheral blood mononuclear cells and colorectal cancer models documented that miR-21 expression decreased following daily curcumin intake, along with subsequent inhibition of key proliferative kinases, such as AKT kinase and the mechanistic (mammalian) target of rapamycin (mTOR) pathway, JNK kinase, and AP-1 transcription factor. This resulted in a reduction in inflammatory response through decreased NF-κB activation, TNF-α synthesis, and IL-6 [3,90]. However, unless miR-21 inhibition or restoration was shown to modify these downstream effects, the available evidence should be interpreted as an association rather than proof that curcumin-induced inhibition of these pathways is miR-21-dependent. Moreover, validation in psoriatic keratinocytes and other dermatologically relevant models remains necessary.

Studies demonstrated that resveratrol significantly reduces miR-21 expression [3]. The effects of resveratrol associated with inhibition of miR-21 expression have been described in cell lines of prostate cancer, pancreatic cancer, glioblastoma, colorectal cancer, and bladder cancer but evidence in cutaneous models is limited [91]. In non-cutaneous experimental platforms (specifically, U251 glioma cell lines and circulating human PBMCs) decreased miR-21 expression has co-occurred with attenuation of pro-inflammatory nuclear transcription factor (NF)-κB and decreased expression of TNF-α, IL-1β, and IL-6. Moreover, resveratrol can reduce the expression of mitogen-activated protein kinase (MEK), JAK kinase, and AP-1 transcription factor [92,93,94]. Current data do not establish that the anti-inflammatory effects of resveratrol in skin are mediated by miR-21. Dedicated functional studies in cutaneous inflammatory models are needed.

Moreover, studies demonstrated that polyphenon-60, containing EGCG as the main component, causes downregulation of miR-21 expression. However, skin-specific validation is still lacking [95]. Studies in humans demonstrated that a diet rich in quercetin has anti-tumor effects [91]. Moreover, in a mouse model, it was proven that quercetin is capable of inhibiting the increase in miR-21 induced by hexavalent chromium [Cr(VI)], a carcinogenic compound, and the associated inhibition of programmed cell death protein 4 (PDCD4) expression in human bronchial epithelial BEAS-2B (BEAS-2B) cells [96]. Notably, these observations were made outside a cutaneous context, and the absence of skin-specific models leaves their relevance to dermatologic systems inferential rather than established. In studies conducted by Zaman and colleagues, it was discovered that genistein inhibits tumor formation through suppression of miR-21 expression in A-498 renal cancer cells and xenografts [97]. Although the data is biologically informative, they derive from renal cancer cells and xenografts, not skin models, and thus should be interpreted as hypothesis-generating rather than directly translatable to cutaneous biology. 

Studies examining the effects of vitamin D on miR-21 expression show a lack of a direct effect of supplementation with cholecalciferol or calcitriol on circulating miR-21 levels [98]. However, there are indirect associations between vitamin D pathways and miR-21 regulation [99,100]. The beneficial effects of vitamin D in psoriasis were confirmed many years ago, but there is no article addressing the effects of vitamin D on miRNA expression profiles in psoriasis. In recent years, the effects of vitamin D have been recognized and explained in terms of its influence on miRNA expression profiles, which contributes to immunosuppression. The significance of vitamin D in balancing proper miR-21 expression has been documented in vascular tissue and osteoblast cultures, whereas direct verification in cutaneous biopsies or primary keratinocytes remains lacking. Regarding the relationship between vitamin D and miRNA expression in psoriasis, assessment of vitamin D levels in plasma is recommended before supplementation, and subsequent, supplementation treatment in cases of deficiency may be considered very important in selected patients [3]. 

In studies on MCF-7 breast cancer cells, it was demonstrated that ALA alone or in combination with EPA and DHA significantly reduces miR-21 expression within 1–3 h of incubation (most strongly in the first hours following exposure) [101]. Nevertheless, the absence of skin-specific experimental data substantially limits the direct translational relevance of these findings, and their applicability to cutaneous biology remains speculative until confirmed in dermatologic models.

Studies examining the Mediterranean diet demonstrate an association between adherence to this diet and levels of various microRNAs. In the study by Fontalba-Romero et al., an association was confirmed between adherence to the Mediterranean diet recommendations and higher plasma miR-590 levels; however, no change in miR-21 levels was found [102]. Such associations have been confirmed for the “Western diet,” rich in sugars and fats. It was demonstrated that following stimulation with high glucose concentration in RAW 264.7 macrophages, there was an “upregulation” in a time-dependent manner of miR-21 [103]. In turn, a prolonged high-fat diet (HFD) increased miR-21 expression in mice and induced obesity. It is worth noting that miR-21 itself is highly expressed in human adipose tissue and correlates positively with BMI [104,105].

Regarding the use of addictive substances, under laboratory conditions, it was confirmed that psychological stress and associated significantly excessive alcohol consumption (referred to as “heavy drinkers,” defined as regular alcohol consumption over the past year of at least 8 standard drinks per week in women and at least 15 standard drinks per week in men) also correlate with excessive miR-21 expression and several of its target genes [106]. Similarly, tobacco smoking and nicotine exposure increase miR-21 expression [107,108]. However, these findings were derived from systemic or behavioral models rather than skin-specific experimental systems, leaving their relevance to cutaneous biology inferential rather than established. Considering the results of Nielsen et al.’s studies, who observed a decrease in circulating plasma miR-21 levels in response to chronic physical exercise, it appears that a sedentary lifestyle may also adversely affect miR-21 signaling [109]. 

In several studies, the therapeutic potential of coenzyme Q10 (CoQ10) in the treatment of diabetes was evaluated. Khalil et al. investigated this issue in an animal model of type II diabetes and demonstrated that CoQ10 supplementation (20 mg/kg intraperitoneally, 3 times per week for 8 weeks) restores glucose/insulin homeostasis, oxidative parameters, cholesterol levels, and expression of miR-33a, miR-21, and sterol regulatory element binding protein 1 (SREBP1) [110]. Taken together, the diabetic data point to a potentially relevant regulatory axis; however, its translational significance for skin biology remains unproven in the absence of dermatologic or cutaneous model systems. 

Although the effects of lycopene on miRNA expression in dermatological diseases have not been described to date, it was investigated in a mouse model of hepatic steatosis: lycopene exhibited protective effects on the liver through the modulation of miR-21 and normalized the downregulation of miR-21 induced by a high-fat diet [111].

### 5.3. miR-146a

MiR-146a is considered a negative regulator of inflammatory status and autoimmune responses. It acts as a suppressor of mRNA transcripts of pro-inflammatory transcription factors and their downstream signaling molecules. This molecule has demonstrated regulatory roles in dermatological diseases such as psoriasis, atopic dermatitis, hidradenitis suppurativa, oral lichen planus, and acne vulgaris [2,112]. In psoriasis, miR-146a regulates inflammatory status and keratinocyte proliferation involved in the pathophysiology of these dermatoses. Both in psoriasis-affected skin and in the serum of patients with active disease, elevated miR-146a expression levels were found. miR-146a reflects keratinocyte sensitivity to IL-17A, acting as a suppressor of skin inflammation induced by psoriasis [3].

In patients with atopic dermatitis, overexpression of miR-146a was also found both in affected skin and serum [2]. Its role in AD pathogenesis is likely associated with inhibition of pro-inflammatory cytokine production in keratinocytes and fibroblasts through sup-pression of various components of the NF-κB pathway. Moreover, through effects on primary human keratinocytes, it leads to reduced expression of pro-inflammatory cytokines C-C motif chemokine ligand 5 (CCL5), IL-8, and ubiquitin D2. MiR-146a is also one of the molecules that shows overexpression in disease-affected skin and in the “perilesional” areas of patients with hidradenitis suppurativa, as confirmed in the Hessam et al. study [113].

In acne vulgaris, miR-146a regulates the inflammatory response induced by *Cutibacterium acnes*. Its increased expression in skin lesions in patients with acne vulgaris was associated with decreased production of IL-6, κB, and TNF-α through inhibition of Toll-like receptor (TLR) 2/interleukin 1 receptor associated kinase 1 (IRAK1)/TNF receptor associated factor 6 (TRAF6)/NF-κB and MAPK pathways [112]. Dietary components significantly modulate miR-146a expression through diverse molecular mechanisms.

In the study by Sun et al., using a Staphylococcus aureus-infected mouse mastitis model, it was demonstrated that selenium inhibits inflammation by inducing microRNA-146a. Dose-dependent selenium supplementation increased expression of this molecule, which in turn led to inhibition of TLR2/6, NF-κB, and MAPK pathways [45]. Importantly, pharmacological inhibition of miR-146a partially reversed the anti-inflammatory effects of selenium, supporting a miR-146a-dependent contribution to selenium-mediated suppression of inflammatory signaling in this model. However, no studies have yet validated this anti-inflammatory miRNA axis in a dermatologic context, so its relevance to skin biology remains speculative.

The effect of the active form of vitamin D (1,25(OH)_2_D_3_) on miR-146a was investigated by Karkeni et al. in a metabolic context. The study showed that vitamin D modulates miR expression (including miR-146a) in adipocytes in vitro and in adipose tissue in vivo by influencing the NF-κB signaling pathway. It was emphasized that expression of, among others, miR-146a was effectively inhibited by pre-incubation with 1,25(OH)_2_D [114].

The effect of elocalcitol, a fluorinated analogue of vitamin D, on miR-146a and its potential involvement in alleviating neuroinflammation and obesity-associated aging has also been described. Through the modulation of miR-146a, elocalcitol prevents the development of microglial cell senescence, whereas miR-146a provides protection against hypothalamic cellular aging induced by a high-fat diet, most likely through inhibition of the TGF/Smad4 pathway [115]. Taken together, this data suggests a biologically meaningful role for vitamin D–miR-146a signaling; however, the absence of skin-based validation limits direct translation to dermatologic contexts.

Quercetin effectively modulates miR-146a, although the currently available studies have been conducted in oncologic rather than dermatologic models. In patients suffering from lung cancer, an increase in miR-146a expression was noted in those consuming more quercetin [116]. The effect of quercetin on miR-146a was also investigated in the context of cell proliferation in human breast cancer cell lines MCF-7 and MDA-MB-231. In the study by Tao et al., quercetin demonstrated an excellent inhibitory effect on cell proliferation in human breast cancer cells, achieved through increased miR-146a expression, subsequently inducing apoptosis via activation of caspase-3 and mitochondria-dependent pathways, as well as inhibiting invasion through reduced EGFR expression [117].

In a dermatological context, quercetin and quercetin-rich plants have shown documented efficacy in experimental treatment of psoriatic lesions. Their mechanism of action involves the inhibition of NF-κB factor, leading to a reduction in inflammatory status and a lowering of pro-inflammatory cytokine levels (TNF-α, IL-6, IL-17) in patient serum [118,119]. Although quercetin-induced upregulation of miR-146a has been described in non-dermatologic models, the reported inhibition of NF-κB and reduction in serum TNF-α, IL-6, and IL-17 in psoriasis-related studies should not currently be considered miR-146a-dependent. Direct demonstration of a quercetin–miR-146a–target axis in cutaneous inflammatory models is still lacking.

### 5.4. miR-155

Overexpression of miR-155 has been described in numerous dermatological conditions, including psoriasis, atopic dermatitis, oral lichen planus, hidradenitis suppurativa, vitiligo, and bullous pemphigoid [2]. miR-155 is classified as a pro-inflammatory miRNA and can stimulate TNF-α production. Additionally, by inhibiting IL-4 expression, it may promote the differentiation of immune responses toward Th1 [120]. It participates in keratinocyte proliferation and differentiation, apoptosis processes, and the regulation of immune responses. In vitiligo, it inhibits genes involved in melanocyte differentiation and activates the JAK-STAT pathway [121]. Several studies have analyzed the role of miR-155 in response to various nutrients present in the human diet.

Quercetin has been reported to reduce miR-155 expression concomitantly with the inhibition of NF-κB activation and lower TNF-α, IL-1, and IL-6 levels as described in the Boesch et al. study by Boesch et al [122]. Although this study was performed in a non-cutaneous murine macrophage model and identified a potentially relevant association between miR-155 downregulation and attenuated NF-κB-related inflammatory signaling, skin-specific experimental data are still lacking. Moreover, because the study did not include functional manipulation of miR-155, it provides associative rather than causal evidence and does not establish that the anti-inflammatory effects of quercetin are miR-155-dependent.

In vitro experiments using healthy monocytic cells (THP-1) incubated with resveratrol at concentrations of 30 and 50 µM for 14 h showed reduced miR-155 expression [123]. This association was also confirmed in an in vivo study of type 2 diabetes patients treated with antihypertensive drugs who took resveratrol-enriched grape extract (8 mg resveratrol) for one year. Resveratrol demonstrated beneficial immunomodulatory effects through re-duction of key pro-inflammatory cytokines and miRNA molecules associated with the regulation of inflammatory responses, including miR-155 [94]. While these findings highlight a potent systemic immunomodulatory cascade, direct verification in cutaneous cell types under inflammatory stimuli is strictly necessary.

The PI3K/AKT pathway is essential for miR-155 inhibition, and it was shown that curcumin significantly reduces phosphorylation of PI3K, p85α, and AKT [124,125]. Moreover, the literature describes reduction in miR-155 expression under the influence of curcumin in salivary gland tumor cells [126], breast cancer cell lines [127,128], extravillous trophoblast cells [129], colorectal cancer [130], and multiple sclerosis [131]. Although curcumin has been shown to suppress miR-155 through the PI3K/AKT pathway in several experimental systems, these findings have largely been generated in non-dermatologic models, and validation in skin-specific systems is still needed.

Apigenin has been shown to suppress lipopolysaccharide-induced miR-155 expression in an in vivo model of murine peritoneal macrophages at 50 mg/kg, apparently through NF-κB blockade; however, despite the mechanistic interest of this axis, the absence of skin-specific experimental systems limits direct extrapolation to dermatologic contexts [132]. 

Given reports on the anti-inflammatory and anti-tumor effects of polyphenols contained in pomegranate fruit (*Punica granatum* L.), studies have also been conducted in this area. Banerjee et al. demonstrated that pomegranate fruit extract induces SHIP-1 expression along with concomitant reduction in miR-155 expression and inhibition of PI3K-dependent AKT phosphorylation in breast cancer cells in vivo and in vitro, however, no skin-specific models were included [133]. 

Vitamin C influences miRNA expression, including miR-155, as confirmed by results published by Kim et al. Daily consumption of a high dose of vitamin C (1250 mg) for 8 weeks resulted in increased anti-aging and anti-atherosclerotic effects through improvement of lipoprotein parameters and reduction of miR-155 expression [134]. In another study, human serum albumin (HSA) nanoparticles enriched with vitamin C reduced miR-155 expression but increased TGF-β1, mothers against decapentaplegic homolog 1 (SMAD1), and SMAD2 expression [135]. Although the cited evidence includes a skin-related wound-healing model, it does not include psoriasis, atopic dermatitis, or hidradenitis suppurativa models. Its relevance to inflammatory dermatoses therefore remains uncertain.

MiR-155 levels decrease following vitamin D administration both in in vitro and in vivo experiments. The anti-inflammatory effect manifests through reduction of NF-κB signaling and p65 subunit [114,136]. The effect of cholecalciferol on reduction of miR-155 expression was also investigated in multiple sclerosis, where a correlation between miR-155-5p and the store operated calcium entry associated regulatory factor (*SARAF*) gene, which plays a role in regulation of store-operated calcium entry channels, was found. It is suggested that the miR-155-5p-SARAF axis hypothesis may be an additional mechanism through which cholecalciferol supplementation can reduce miR-155 expression [137]. Taken together, the reported effects of vitamin D-, cholecalciferol-, related interventions on miR-155 remain preliminary. Validation in disease-relevant models of psoriasis, atopic dermatitis, or hidradenitis suppurativa is lacking.

Polyunsaturated fatty acids modulate miRNA expression independently of the presence/absence of inflammatory stimuli. It has been reported that excessive miR-155 expression is observed in response to ω-3 PUFA (docosahexaenoic acid) supplementation in endothelial cells under inflammatory conditions. In an inflammatory environment, both docosahexaenoic acid (ω-3 PUFA) and arachidonic acid (ω-6 PUFA) activate TLR signaling and macrophage differentiation [138,139].

In the Marques-Rocha et al. study, the effect of oleic acid on increased miR-155 expression in monocytes compared to DHA was described [140]. Importantly, these fatty acid–miR-155 relationships were investigated exclusively in non-cutaneous experimental systems, and no skin-specific models were included.

### 5.5. miR-203

Studies have demonstrated that the mature form of miR-203 plays a key role in neurodegenerative diseases, metabolic disorders, and cancers [141,142]. This molecule participates in metabolic adaptations of adipose tissue due to its browning-promoting effects on white adipose tissue and differentiation of brown adipocytes [143]. In cancers, miR-203 levels are “downregulated,” which is associated with inhibition of cell proliferation. Such an association has been described in melanoma, esophageal squamous cell carcinoma, hematological malignancies, prostate cancer cell lines, and pancreatic adenocarcinoma [142]. MiR-203 is also a skin-specific miRNA, overexpressed in keratinocytes of skin lesions in psoriasis patients. Increased miR-203 expression plays an important role in keratinocyte differentiation and psoriasis pathogenesis, as well as the development of atopic dermatitis in children and lichen planus [2]. In psoriasis, miR-203 overexpression is associated with the following factors: increased STAT3 protein expression, protein kinase C expression, in-creased transcription of pro-inflammatory cytokines such as TNF-α, IL-8, and IL-24, and decreased suppressor of cytokine signaling 3 (SOCS3) protein expression [144,145,146,147]. Moreover, the overexpression of miR-203 was reported in the serum of children with atopic dermatitis aged 6 months to 6 years [148]. miR-203 expression can be modulated by diet type, translating into adaptive and pathophysiological changes in the body.

In a study published by Takahashi et al., the effects of a low-protein diet on miR-203 expression in rats were investigated. Six-week-old male rats were fed either a control diet (20% casein) or a low-protein diet (5% casein) for two weeks, and their livers were analyzed using DNA microarrays and miRNA microarrays. The low-protein diet showed decreased miR-203 expression and “up-regulation” of the β-subunit of hydroxy-acyl-CoA dehydrogenase (*Hadhb*), a likely target molecule for miR-203. Increased *Hadhb* expression secondarily led to enhanced β-oxidation of fatty acids as a compensatory mechanism for protein deficiency [142].

In turn, in the Ramzan et al. study, the effects of a high-protein diet (1.6 g/kg body weight/day protein) on selected miRNA expression were evaluated over 10 weeks in men over 70 years of age compared to a group consuming recommended daily protein intake (0.8 g/kg body weight/day). In the high-protein diet group, reduced expression of five miRNAs, including miR-203 and miR-125b, was found [149].

Dietary changes can also influence miR-203 expression. In the Li et al. study, it was demonstrated that switching from a high-fat diet to a high-carbohydrate diet inhibits expression of the mature form of miRNA (miR-203) in rodents. miR-203 overexpression inhibits the maturation of pancreatic β-cells “at the neonatal stage” and, under high-fat diet conditions, leads to their dysfunction [141]. Moreover, it was shown that a unique combination of chemo-protective fish oil (containing EPA and DHA) and pectin (fermented to butyrate in the colon) could modify a subset of mucosal miRNAs—miR-19b, miR-26b, and miR-203. Increased miR-203 expression inhibits the TCF4 transcription factor and the Wingless-related integration site (Wnt)/β-catenin pathway, reducing potentially neoplastic proliferation [150].

### 5.6. miR-125b

Mature miR-125b arises from two precursors, miR-125b-1 and miR-125b-2. This molecule participates in multiple processes, including the inhibition of osteoblast differentiation, the inhibition of hepatocellular carcinoma metastasis, and the regulation of neoplastic cell proliferation and lipogenesis through the modulation of target gene expression [151]. miR-125b expression has also been associated with obesity in mice and humans [151]. In dermatology, its decreased expression has been found in patients with plaque psoriasis and hidradenitis suppurativa. In these dermatoses, its role is linked to TNF-α inhibition and the regulation of keratinocyte differentiation and proliferation through inhibitory effects on fibroblast growth factor receptor 2 (FGFR2) [152]. The impact of dietary components on miR-125b expression from a nutrigenomics pers-pective primarily encompasses modulation of this microRNA through macronutrient proportions (as previously described in the miR-203 section, a high-protein diet contributes to reduced miR-125b expression) [149], as well as specific bioactive plant compounds and lipids.

It has been demonstrated that eicosapentaenoic acid (EPA) reduces lipopolysaccharide-induced inflammatory response in RAW264.7 murine macrophage lines (serving as an in vitro immune cell surrogate rather than a skin model) through the modulation of cellular cytokine production and reduction in miR-125b-5p expression. miR-125b-5p has the capacity to block cAMP response element-binding protein (CREB), whose overexpression reduces the inflammatory status, likely through the modulation of the peroxisome proliferator-activated receptor gamma coactivator 1 alpha (PGC-1α)/NF-κB pathway. Through this modulatory effect of EPA on the miR-125b-5p/CREB axis, control of gene transcription and, consequently, modulation of pro-inflammatory mediator production are achieved [153].

Among polyphenols with documented potential effects on miR-125b expression is quercetin. A diet enriched with quercetin increased miR-125b expression in a laboratory mouse study conducted by Boesch-Saadatmandi et al. [154]. In turn, in the study by Wein et al., a decrease in the expression of numerous miRNAs, including miR-125b, was described in the liver of mice fed quercetin. The ninefold reduction in hepatic miRNA miR-125b-3p was accompanied by significant induction of gamma-glutamyl hydrolase (GGH) mRNA [155].

Another polyphenol is resveratrol. It has been shown that resveratrol increases miR-125b-5p expression through improvement in its stability in colorectal cancer patients, subsequently inhibiting the TRAF6-induced signaling pathway in a dose- and time-dependent manner. Both elevated miR-125b-5p expression and reduced TRAF6 expression inhibited colorectal cancer cell metastasis. Therefore, it was proposed that resveratrol may exert inhibitory effects on colorectal cancer metastasis through promotion of the miR-125b-5p/TRAF6 signaling axis [156]. In contrast, in studies conducted by Venkatadri et al. regarding breast cancer, resveratrol significantly contributed to reduced miR-125b expression (more than a fivefold reduction) while simultaneously promoting apoptosis and pathways that inhibit tumor development. The results of both these studies confirm both the effect of resveratrol on miR-125b and the capacity for pleiotropic modulation by this molecule through effects on both pro- and anti-apoptotic genes [157]. Beyond oncological diseases, the effects of resveratrol on follicular fluid (FF) miRNAs were investigated in older women with low ovarian reserve receiving resveratrol supplementation for three months before in vitro fertilization (IVF) cycles. Resveratrol significantly reduced the expression of several miRNAs, including miR-125b-5p [158]. Importantly, despite the growing evidence that polyphenols can modulate miR-125b expression in experimental, clinical, and oncological settings, no unequivocal skin-specific models have been established, limiting direct extrapolation of these findings to cutaneous biology.

### 5.7. miR-24

MiR-24 expression is altered in various types of cancers at different stages of tumorigenesis. It has also been demonstrated that, depending on cellular context, miR-24 functions as either a tumor suppressor (TS) or a tumor-promoting factor, influencing in-creased expression of oncogenes such as cellular Myc protooncogene protein (c-Myc), B cell lymphoma 2 (BCL2), and hypoxia inducible factor 1 (HIF1) [159]. In the dermatological context, microRNA-24-1-5p promotes autophagy and the apoptosis of malignant melanoma cells through ubiquitin D regulation [160]. Variable miR-24 expression has been described in numerous systemic diseases, including cardiovascular disorders, neurological diseases, gastroenterological diseases, diabetes, rheumatoid arthritis, viral infections [159], and dermatoses [2]. In atopic dermatitis, increased serum miR-24 expression positively correlated with platelet factor 4 (PF-4) plasma concentration and thymus and activation-regulated chemokine (TARC) levels, which may indicate a role for platelet activation in the pathogenesis of atopic dermatitis. The potential role attributed to miR-24 is associated with suppression of interferon-gamma expression and, consequently, Th1 response [161]. Conversely, in psoriasis patients, decreased hsa-miR-24-3p expression in blood has been described compared to the control group [162]. Decreased miR-24 expression has also been described in peripheral blood leukocytes of patients with hidradenitis suppurativa [163].

Reports on direct effects of nutrigenomic components on miR-24 are limited. Anthocyanins from black raspberries (BRB) show the most documented effects on miR-24, particularly in chemoprevention of colorectal cancer. In a mouse model, BRB supplementation for 9 weeks significantly increased miR-24-1-5p expression in colon tissue, subsequently suppressing β-catenin expression, one of the key signaling elements in colorectal cancer development and progression. Increased miR-24-1-5p expression indirectly translated into reduced cell proliferation, migration, and survival. The authors suggest that given the above, miR-24-1-5p may constitute a novel chemopreventive and therapeutic strategy for β-catenin signaling-driven colorectal cancer [164].

Effects of diet type and obesity on miR-24 expression have also been observed. In a HepG2 cell line subjected to conditions mimicking hyperlipidemia and in the livers of mice with diet-induced and genetically determined obesity, miR-24 up-regulation was found. miR-24 overexpression was associated with suppression of Scavenger Receptor B1 (SR-B1) expression, a glycoprotein mediating selective uptake of cholesterol esters from lipo-proteins under in vivo and in vitro conditions. This resulted in suppression of HDL uptake and steroidogenesis in steroidogenic cells, reduced triglyceride levels, and lipid accumulation. Furthermore, miR-24 overexpression reduced the expression of some genes in-volved in lipogenesis and increased the expression of cholesterol synthesis genes [165]. Interestingly, miR-24 (specifically miR-24-1-5p) appears to affect bone mineralization at birth through a maternal high-protein diet and reduces bone mass in offspring throughout life due to decreased osteoblast maturation [166]. Taken together, although accumulating evidence links dietary factors to miR-24 regulation, these findings are still largely derived from non-cutaneous systems, underscoring the need for further studies in skin-specific and dermatological models. 

### 5.8. miR-223

MiR-223 is one of the key immunometabolic regulators that play a role in lipid homeo-stasis. It controls intracellular cholesterol levels by directly suppressing genes involved in cholesterol biosynthesis (3-hydroxy-3-methylglutaryl-CoA synthase 1 [*HMGCS1*] and sterol 27-hydroxylase [*SC4MOL*]) and cholesterol uptake (scavenger receptor class B member 1) in hepatocytes and coronary artery endothelial cells [167]. Apart from cholesterol metabolism, miR-223 inhibits the TLR4-NFκB pathway, leading to reduced production of pro-inflammatory cytokines in macrophages activated by LPS19, likely resulting from direct effects on IKB kinase α [IKKα] and reduction in non-canonical NFκB activation [167]. miR-223 is also a negative regulator of NLRP3 inflammasome activation through direct suppression of NLRP3 expression and reduced NLRP3-dependent IL-1β induction in primary neutrophils [167].

In patients with atopic dermatitis, significantly elevated serum miR-223 expression levels are observed, which is associated with platelet activation. Furthermore, this level positively correlates with symptom severity in atopic dermatitis and with serum TARC, which is a potent chemoattractant for Th2 helper cells, acting through chemokine receptor CC2. Similarly, in patients with hidradenitis suppurativa, increased miR-223 expression in skin lesions has been observed. In this dermatological condition, it is proposed that miRNA overexpression is caused by significant neutrophil accumulation in inflamed tissue [168]. miR-223 is also one of the molecules showing overexpression in the serum of plaque psoriasis patients, as confirmed by studies by Higashi and Madaan [169,170]. Conversely, in inflammatory dermatoses, significantly reduced miR-223 expression was found in the skin in early-stage mycosis fungoides, with further reduction at advanced disease stages [171].

In a study published by Shan et al., the effects of theabrownin (TB) from pu-erh tea extract on rats with metabolic syndrome were investigated. Treatment with theabrownin containing extract significantly improved metabolic syndrome markers, including dyslipidemia, insulin resistance, and inflammation. This improvement was associated with normalization of specific miRNAs (miR-125b-5p, miR-148b-3p, miR-1247-5p, and miR-223-3p_R + 2), which activated the PI3K/AKT/FOXO signaling pathway, subsequently modulating key genes involved in glycolipid metabolism (*SREBP-1C*, phosphoenolpyruvate carboxykinase [*PEPCK]*, PGC-1α, and glucose 6 phosphatase catalytic subunit [*G6pc*]) [172].

Dietary modifications have also demonstrated effects on miR-223 expression: a high-fructose diet (decreased expression) [173] or a diet high in dairy products (increased expression) [174]. In the case of a diet rich in TFA, the study by Desagne et al. did not confirm a significant unidirectional effect of such modification on HDL-carried miR-223-3p levels. However, changes in HDL-miR-223-3p levels showed a negative correlation with changes in HDL cholesterol levels after a diet rich in industrial trans fatty acids (rs = −0.82; *p* = 0.007) and a positive correlation with changes in C-reactive protein levels after a diet rich in ruminant-derived trans fatty acids (rs = 0.75; *p* = 0.020). Compared to a control diet, changes in HDL-miR-135a-3p levels positively correlated with changes in total triglyceride (TG) levels after a diet rich in industrial trans fatty acids (rs = −0.82; *p* = 0.007) and with changes in LDL-TG levels after a diet rich in ruminant-derived trans fatty acids (rs = 0.83; *p* = 0.005). The authors suggest that the variability in HDL-carried miRNA responses after TFA consumption, through association with changes in cardiovascular disease risk factors, may reflect physiological changes in HDL functions [175]. Although a growing body of evidence links miR-223 to systemic immunometabolic regulation and demonstrates its sensitivity to dietary modulation, this data derives almost exclusively from hepatic, vascular and circulating compartments rather than from skin-specific systems. Consequently, the relevance of diet-induced changes in miR-223 to cutaneous immune homeostasis and inflammatory dermatoses remains largely inferential, underscoring the need for mechanistic studies in skin cells and in vivo dermatologic models. 

The modulation of specific microRNA expression profiles by dietary bioactives is systematically summarized in Table 2.

**Table 1:** Expression of selected miRNAs in inflammatory skin diseases. The reported expression patterns are derived from heterogeneous studies that differ in disease phenotype, disease activity, tissue or biofluid source, cell type, analytical platform, normalization strategy, and concomitant treatment. Accordingly, these miRNAs should currently be regarded as candidate disease-associated markers rather than validated diagnostic, prognostic, or treatment-selection biomarkers.

Disease	miRNA–Upregulated	miRNA–Downregulated	References
Psoriasis	miR-31; miR-21; miR-146a; miR-203; miR-223	miR-125b, miR-24-3p	miR-31: [1,2,80]; miR-21: [2,80,83,84]; miR-146a: [1,2,3,80]; miR-203: [2,80,144,146]; miR-223: [80,169,170]; miR-125b: [80,84,152]; miR-24-3p: [80,162]
Atopic dermatitis	miR-21; miR-146a; miR-155; miR-203; miR-24; miR-223	miR-125b	miR-21: [2,80,84]; miR-146a: [2,80]; miR-155: [2,80]; miR-203: [2,80,148]; miR-24: [2,80,161]; miR-223: [80,168]; miR-125b: [2,80]
Hidradenitis suppurativa	miR-31; miR-146a; miR-155; miR-223	miR-125b; miR-24	miR-31: [113] miR-146a: [113]; miR-155: [113]; miR-223: [113,168]; miR-125b: [113]; miR-24: [163]

**Table 2:** Dietary factors reported to modulate selected microRNAs: evidence category and translational relevance to inflammatory skin diseases. No studies directly evaluating dietary factor-induced modulation of miRNA expression in patients with psoriasis, atopic dermatitis, or hidradenitis suppurativa were identified among the studies summarized in this table. Category 1—Direct human inflammatory skin-disease evidence; Category 2—Skin-specific preclinical evidence; Category 3—Indirect non-cutaneous evidence; Category 4—Mechanistic/speculative association.

miRNA Molecule	Nutrient Compound/Diet Modification	Type of Regulation	Study Aim	Evidence Category	Interpretation for Inflammatory Skin Diseases	Literature
miR-31	Iron	↑	Patients with head and neck squamous cell carcinoma	Category 3	Associations were observed in a non-cutaneous oncological cohort. Their relevance to miR-31 regulation in inflammatory skin disease is unknown.	[81]
	Vitamin C	↑				
	Total sugar	↓				
	Cholesterol	↓				
	Vitamin B9	↓				
	*Trans* fatty acids (iTFAs and rTFAs)	↓	HDL-associated miRNAs in healthy men	Category 3	The observation concerns circulating HDL-associated miRNA and cannot be extrapolated to cutaneous miR-31 expression or inflammatory dermatoses.	[82]
miR-21	Curcumin	↓	Colorectal cancer cells and murine xenograft models	Category 3	Curcumin-associated miR-21 downregulation was demonstrated in colorectal cancer models. Its relevance to psoriatic keratinocytes, AD, or HS has not been established.	[89]
	Resveratrol	↓	Cancer cell lines, including glioma, prostate, pancreatic, colorectal, and bladder cancer models; PBMCs in cardiometabolic disease	Category 3	Resveratrol can modulate miR-21 in non-cutaneous systems. A causal effect on miR-21-dependent inflammatory pathways in human skin disease remains unproven.	[91]
	EGCG (polyphenon-60)	↓	Breast and prostate cancer cell lines	Category 3	Findings derive from oncological models and do not demonstrate regulation of miR-21 in inflammatory skin disease.	[91]
	Quercetin	↓	BEAS-2B bronchial epithelial cells and xenograft mouse model	Category 3	Quercetin–miR-21 regulation was reported in bronchial epithelial and cancer-related models. Skin-specific validation is lacking.	[96]
	Genistein	↓	A-498 renal cancer cells and xenograft models	Category 3	This association was observed in renal cancer models; its relevance to inflammatory dermatoses is speculative.	[91]
	Alpha-linolenic acid (ALA)	↓	MCF-7 breast cancer cells	Category 3	Regulation was observed in breast cancer cells and has not been replicated in skin-derived cells or dermatological models.	[101]
	High glucose	↑	RAW 264.7 macrophages	Category 3	The finding supports a possible link between metabolic inflammation and miR-21 but does not demonstrate an effect in cutaneous inflammation.	[88]
	High-fat diet	↑	Adipose tissue in diet-induced obese mice	Category 3	High-fat diet-related changes in adipose-tissue miR-21 cannot be assumed to reflect miR-21 regulation in skin.	[88]
	Excessive alcohol consumption	↑	Peripheral blood from non-smoking social drinkers	Category 3	The relationship is observational and based on circulating blood miRNA; its dermatological relevance remains unknown.	[106]
	Nicotine exposure	↑	Oesophageal cancer cells	Category 3	The finding was obtained in an oncological epithelial model and is not evidence of a causal role in inflammatory skin disease.	[107]
	Coenzyme Q10	↓	Pancreatic tissue in diabetic rats	Category 3	This observation in pancreatic tissue requires validation in skin-specific systems.	[110]
	Lycopene	↓	Liver tissue in high-fat-diet-fed mice	Category 3	Evidence concerns hepatic metabolism in mice and does not establish a dermatological miR-21 effect.	[111]
miR-146a	Selenium	↑	*Staphylococcus aureus*-infected mouse mastitis model	Category 3	Selenium-induced miR-146a expression was observed in mammary tissue, not skin. Relevance to cutaneous inflammatory disease remains unconfirmed.	[45]
	Vitamin D	↓	Adipocytes in vitro and adipose tissue in vivo	Category 3	The relationship was evaluated in adipose tissue and cannot establish vitamin D-mediated miR-146a regulation in inflammatory dermatoses.	[115]
	Elocalcitol	modulation	High-fat-diet-fed mice; microglial model	Category 3	The effect is reported in a metabolic/neuroinflammatory context and requires skin-specific confirmation.	[115]
	Quercetin	↑	Lung cancer tissue and breast cancer cell lines	Category 3	Quercetin-related miR-146a modulation was reported in oncological models. It does not show a nutrient–miRNA effect in inflammatory skin disease.	[116,117]
miR-155	Quercetin	↓	LPS-stimulated RAW 264.7 macrophages stimulated with lipopolysaccharide	Category 3	The miR-155 effect was demonstrated in macrophages, without validation in skin-derived cells or patients with inflammatory dermatoses.	[122]
	Resveratrol	↓	THP-1 monocytes; PBMCs from patients with type 2 diabetes, hypertension, and coronary artery disease	Category 3	The association concerns circulating immune cells in cardiometabolic disease; its relevance to skin inflammation is unconfirmed.	[94]
	Curcumin	↓	LPS-treated macrophages and mice; additional non-cutaneous cell lines and PBMCs	Category 3	Curcumin may alter miR-155 in systemic inflammatory or oncological contexts, but evidence in inflammatory skin disease is lacking.	[126,127,128,129,130,131]
	Apigenin	↓	Macrophage-based experimental models	Category 3	The reported interaction derives from immune-cell systems and requires confirmation in skin-derived or dermatological models.	[176]
	*Punica granatum* L.	↓	Breast cancer cells and animal models	Category 3	Findings in breast-cancer systems cannot be directly translated to inflammatory skin disease.	[133]
	Vitamin C	↓	HDL fraction from healthy subjects; HSA nanoparticle-based mouse wound-healing model	Category 2	A cutaneous wound-healing model supports biological plausibility, but it is not a model of psoriasis, AD, or HS; clinical relevance to inflammatory dermatoses remains unestablished.	[134,176]
	Vitamin D	↓	Multiple-sclerosis cohorts	Category 2	Vitamin D-related modulation was not studied in inflammatory dermatoses. Translation to psoriasis, AD, or HS remains uncertain.	[3,137]
	DHA/ω-3 PUFA	↑/modulation	Human endothelial cells under inflammatory stimulation	Category 3	The evidence comes from vascular endothelial cells and does not demonstrate miR-155 regulation in skin inflammation.	[176]
	Oleic acid	↑/modulation	Monocytes, macrophages, and LPS-activated macrophages	Category 3	Observations in monocyte/macrophage systems require validation in relevant dermatological models.	[140]
miR-203	Low-protein diet	↓	Liver tissue of Wistar rats	Category 3	The finding concerns hepatic metabolic adaptation and cannot be extrapolated to epidermal miR-203 biology.	[142]
	High-protein diet	↓	Circulating miRNA in older men	Category 3	Systemic circulating-miRNA data do not demonstrate an effect on epidermal miR-203 or inflammatory skin disease.	[149]
	Dietary switch from high-fat to high-carbohydrate diet	↓	Mouse model	Category 3	As the relevant tissue is not dermatological, the association remains indirect for inflammatory skin diseases.	[141]
miR-125b	EPA	↓	LPS-stimulated RAW 264.7 macrophages	Category 3	EPA-related regulation in macrophages is biologically informative but has not been validated in skin-derived cells or inflammatory dermatoses.	[153]
	Quercetin	↑	Mouse liver	Category 3	Hepatic miR-125b regulation does not establish relevance to cutaneous inflammation.	[154]
		↓	Wistar rat liver	Category 3	The direction of effect differs across hepatic models, further limiting extrapolation to inflammatory skin disease.	[155]
	Resveratrol	↑	Colorectal cancer cells and tissues	Category 3	This interaction was observed in colorectal cancer models and remains unvalidated in skin.	[156]
		↓	Breast cancer cells and follicular fluid from older women	Category 3	The divergent finding illustrates context-dependent regulation and cannot be used to predict cutaneous effects.	[157,158]
miR-24	Black raspberry anthocyanins	↑	Colonic tissue in azoxymethane/dextran sulfate sodium-treated mice	Category 3	The study concerns colonic inflammation/carcinogenesis and does not demonstrate relevance to skin inflammation.	[164]
	Diet-induced obesity	↑	HepG2 cells and liver tissue from diet-induced and genetic obesity mouse models	Category 3	The association is metabolic/hepatic and requires validation in inflammatory skin disease.	[165]
miR-223	theabrownin (from Pu-erh tea)	↓	High-fat-diet-induced metabolic syndrome in rats	Category 3	This is a systemic metabolic model; it does not establish cutaneous miR-223 modulation.	[172]
	High-fructose diet	↓	Mouse liver	Category 3	Hepatic findings do not demonstrate a link to miR-223 regulation in skin disease.	[173]
	high-diary diet	↑	Healthy human participants	Category 3	The reported association is systemic and observational; its relevance to inflammatory dermatoses is unknown.	[174]

Note: ↑ increased expression; ↓ decreased expression.

## 6. Limitations 

A critical consideration in evaluating the therapeutic potential of dietary bioactives is their variable and often low oral bioavailability. Many of the most potent nutrigenomic modulators discussed, particularly polyphenols such as curcumin, quercetin, and resveratrol, exhibit poor aqueous solubility, rapid gastrointestinal metabolism, and high systemic clearance [177,178]. Consequently, the high concentrations frequently utilized in in vitro models to induce miRNA remodeling do not readily translate to target cutaneous tissues following standard dietary intake. To overcome this significant limitation, current translational research is increasingly focusing on innovative delivery systems—such as liposomes, and solid lipid nanoparticles—which substantially enhance the stability, absorption, and targeted delivery of these compounds. Acknowledging these pharmacokinetic constraints is essential for accurately bridging theoretical nutrigenomic mechanisms with effective, reproducible clinical interventions [179,180]. 

Furthermore, most of available studies investigating isolated bioactive components—such as individual polyphenols. While it provides crucial insights into specific molecular pathways, this single-compound approach carries inherent limitations in nutrigenomic research. In real-world human nutrition, dietary factors are consumed not in isolation, but as part of a complex food matrix. Consequently, single-compound studies often overlook the synergistic, additive, or antagonistic interactions occurring among multiple phytochemicals, which collectively modulate biological efficacy. Furthermore, a significant portion of in vitro and animal models utilizing single compounds rely on supra-physiological concentrations. These high doses do not accurately reflect the actual bioavailability, systemic pharmacokinetics, or extensive metabolic transformations—including gut microbiota processing—associated with standard nutritional intake. Therefore, while single-compound data remain essential for baseline mechanistic proof-of-concept, their translational validity to human clinical outcomes is limited, underscoring the need for future research to transition toward whole-food matrices and multi-compound synergistic models.

In addition, despite the therapeutic promise of miRNA modulation, accurate miRNA detection faces some methodological constraints. Technical challenges stem from their short sequence length (19–25 nt), low endogenous abundance, and high sequence homology within families [181,182]. Pre-analytical factors, such as sample handling, RNA extraction efficiency, and hemolysis, can significantly distort baseline levels [182,183]. Additionally, analytical platforms (RT-qPCR, NGS, microarrays) introduce distinct platform-specific biases, while the lack of universally accepted endogenous reference genes complicates normalization. Standardizing pre-analytical protocols and normalization methods is essential for reproducible translation into clinical dermatology [181,182,183].

Furthermore, a key limitation of current cutaneous nutrigenomic research is that a substantial proportion of available mechanistic data on nutrient–miRNA interactions derive from non-dermatological systems, including oncological, hepatic, and vascular models. While these findings provide valuable proof-of-concept for metabolic gene regulation, tissue-specific regulatory networks and target gene accessibility differ substantially within the stratified cutaneous microenvironment. Therefore, non-cutaneous evidence must be interpreted strictly as hypothesis-generating until directly validated in human epidermal keratinocytes, dermal fibroblasts, or cutaneous in vivo models. Most human dietary studies demonstrate cross-sectional correlations with circulating miRNAs rather than proven causal mechanisms. Longitudinal interventional trials with paired tissue and serum sampling are required to confirm direct cutaneous epigenetic remodeling.

## 7. Conclusions

Individual dietary macro- and micronutrients, as well as selected bioactive compounds, have been reported to influence microRNA expression in experimental and selected human studies. Identification of disease- and phenotype-associated miRNA profiles may help generate hypotheses for future individualized supportive approaches. However, their use to guide dietary interventions has not yet been validated in prospective clinical studies and is not ready for routine dermatological practice and prescriptive personalized dietary protocols based on individual miRNA profiles. 

Contemporary approaches to inflammatory skin diseases increasingly incorporate the concept of personalized medicine, in which microRNAs can additionally serve as diagnostic, prognostic, and predictive biomarkers. microRNAs fulfill most of the criteria required for an ideal biomarker, such as availability, high specificity, and sensitivity [184]. Given the above premises, investigating the potential effects of diet on microRNA expression in inflammatory skin diseases appears to be not only scientifically justified but also extremely relevant from a clinical perspective. It may contribute to a better understanding of molecular mechanisms linking nutrition to inflammatory skin diseases and may support the development of testable dietary hypotheses. Whether miRNA-informed dietary modification can provide safe, effective, and clinically meaningful benefit beyond conventional treatment remains unknown [3].

## Data Availability

No new data were created or analyzed in this study. Data sharing is not applicable to this article.

## Abbreviations

The following abbreviations are used in this manuscript:

AA	arachidonic acid
AD	atopic dermatitis
ADAM17	a disintegrin and metalloproteinase 17
Akt	Protein kinase B
ALA	alpha linolenic acid
AP-1	Activator Protein 1
ApoB	Apolipoprotein B
Bcl-2	B cell lymphoma 2
BEAS-2B	Human bronchial epithelial cell line BEAS 2B
BRAF	v Raf murine sarcoma viral oncogene homolog B
c-Myc	Cellular Myc proto oncogene protein
CAD	Coronary artery disease
CAT	Catalase
CCL5	C C motif chemokine ligand 5
COPD	Chronic obstructive pulmonary disease
CoQ10	Coenzyme Q10
COX	Cyclooxygenase
CREB	cAMP response element binding protein
CRP	C reactive protein
DHA	Docosahexaenoic acid
dROM	Derivatives of reactive oxygen metabolites
EGCG	Epigallocatechin gallate
EPA	Eicosapentaenoic acid
ERK	Extracellular signal regulated kinase
FBG	Fasting blood glucose
FGFR	Fibroblast growth factor receptor
FOXO	Forkhead box O (transcription factor family)
G6pc	Glucose 6 phosphatase catalytic subunit
GGH	Gamma glutamyl hydrolase
GPx	Glutathione peroxidase
GSH	Glutathione synthetase
Hadhb	Beta subunit of hydroxyacyl CoA dehydrogenase
HAT	Histone acetyltransferases
HDAC	Histone deacetylases
HDL	High density lipoprotein
HIF1	Hypoxia inducible factor 1
HMGCS1	3 hydroxy 3 methylglutaryl CoA synthase 1
HS	Hidradenitis suppurativa
HSA	Human serum albumin
hs-CRP	high-sensitivity C-reactive protein
IHS4	International Hidradenitis Suppurativa Severity Score System
IKB	Inhibitor of kappa B
IKK	IKB kinase
IL	Interleukin
iNOS	Inducible nitric oxide synthase
IRAK1	Interleukin 1 receptor associated kinase 1
JAK/STAT	Janus kinase/Signal Transducer and Activator of Transcription
JNK	c Jun N terminal kinase
LDL	Low density lipoprotein
LPS	Lipopolysaccharide
MAPK	Mitogen activated protein kinase
MCF-7	Human breast adenocarcinoma cell line MCF 7
MDA	Malondialdehyde
MEK	Mitogen activated protein kinase kinase (MAPK/ERK kinase)
miRNA	microRNA
mTOR	Mechanistic (mammalian) target of rapamycin
MUFA	Monounsaturated fatty acids
MyD88	Myeloid differentiation primary response 88
NADPH	Nicotinamide adenine dinucleotide phosphate (reduced form)
NF-κB	Nuclear factor kappa light chain enhancer of activated B cells
NLRP3	NOD-, LRR- and pyrin domain-containing protein 3
NRAS	Neuroblastoma RAS viral oncogene homolog
Nrf2	Nuclear factor erythroid derived 2
PASI	Psoriasis Area and Severity Index
PDCD4	Programmed cell death protein 4
PEPCK	Phosphoenolpyruvate carboxykinase
PF-4	Platelet factor 4
PG	Prostaglandin
PGC-1α	Peroxisome proliferator activated receptor gamma coactivator 1 alpha
PI3K	Phosphoinositide 3 kinase
PPP6C	Catalytic subunit of protein phosphatase 6
PTEN	Phosphatase and tensin homolog
PUFA	Polyunsaturated fatty acids
SARAF	Store operated calcium entry associated regulatory factor
SC4MOL	Sterol C4 methyl oxidase like (sterol C4 methyl oxidase)
SCORAD	SCORing Atopic Dermatitis
SIRT	Sirtuin
SMAD	Mothers against decapentaplegic homolog (SMAD protein family)
SOCS3	Suppressor of cytokine signaling 3
SOD	Superoxide dismutase
SR-B1	Scavenger receptor class B type 1
SREBP	Sterol regulatory element binding protein
STK	Serine/threonine protein kinase
TACE	Tumor necrosis factor α converting enzyme
TARC	Thymus and activation regulated chemokine
TCF	Transcription factor
Teff	Effector T cell
TFA	Trans fatty acid
TGF-β1	Transforming growth factor beta 1
Th	T helper
THP-1	Human monocytic leukemia cell line THP 1
TIMP-3	Tissue inhibitor of matrix metalloproteinase 3
TLR	Toll like receptor
TMAO	Trimethylamine N oxide
TNF-α	Tumor necrosis factor alpha
TRAF	TNF receptor associated factor
Treg	Regulatory T cell
TrxR	Thioredoxin reductase
Wnt	Wingless-related integration site
